# In situ casting of rice husk ash in metal organic frameworks induces enhanced CO_2_ capture performance

**DOI:** 10.1038/s41598-020-77213-9

**Published:** 2020-11-19

**Authors:** Debashis Panda, Chanchal Saini, E. Anil Kumar, Sanjay Kumar Singh

**Affiliations:** 1 Discipline of Mechanical Engineering, Indian Institute of Technology Indore, Simrol, Indore, Madhya Pradesh 453552 India; 2 Discipline of Chemistry, Indian Institute of Technology Indore, Simrol, Indore, Madhya Pradesh 453552 India; 3 Department of Mechanical Engineering, Indian Institute of Technology Tirupati, Tirupati, Andhra Pradesh 517506 India

**Keywords:** Carbon capture and storage, Metal-organic frameworks

## Abstract

Incorporation of rice-husk-ash (RHA), an agricultural waste, in situ during the synthesis of MIL-101(Cr) resulted in a significant improvement in the CO_2_ adsorption properties over the synthesized RHA-MIL-101(Cr). The newly synthesized RHA-MIL-101(Cr) composite exhibited an enhancement of 14–27% in CO_2_ adsorption capacity as compared to MIL-101(Cr) at 25 °C and 1 bar. The content of RHA incorporated in RHA-MIL-101(Cr) fine tuned the CO_2_ capture performance to achieve high working capacity (0.54 mmol g^−1^), high purity (78%), superior CO_2_/N_2_ selectivity (18) and low isosteric heat of adsorption (20–30 kJ mol^−1^). The observed superior CO_2_ adsorption performance of RHA-MIL-101(Cr) is attributed to the fine tuning of textural characteristics—enhancement of 12–27% in BET surface area, 12–33% in total pore volume and 18–30% in micropore volume—upon incorporation of RHA in MIL-101(Cr).

## Introduction

In the modern era, the uphill global energy demand is still mostly satisfied by the combustion of fossils. Worldwide, the unrestricted emission of anthropogenic CO_2_ from the combustion of fossil fuels is the primary contributor to global warming^1–3^. Stationary point sources such as thermal power plants^3^, cement industries^4^ and distributed sources such as transport sectors^5,6^ are mainly responsible for large CO_2_ emission. Based on the Intergovernmental Panel on Climate Change (IPCC) report, the gross emission of CO_2_ from fossil fuels was totaled to about 23.5 Gt CO_2_ year^−1^, in the year 2000^4^. It is also estimated that there will be a rise in CO_2_ emission from 29 to 44 Gt CO_2_ (8–12 GtC) per year in 2020, and from 23 to 84 Gt CO_2_ (6–23 GtC) per year in 2050^4^. This indeed presents a scary scenario of climate change, and hence extensive efforts are being made to develop efficient methodologies to reduce carbon footprint. In this direction, carbon capture and sequestration (CCS) methodologies such as chemical absorption, membrane separation, adsorption, and cryogenic distillation have shown promising outlook to address this global issue appropriately^7^. Among several CCS methodologies, adsorption-driven processes for CO_2_ capture have received extensive attention due to low capital and operating cost, low energy penalty and smaller plant foot prints^7^. Hence, various porous adsorbents, such as mesoporous silica^8^, activated carbon (AC)^9^, zeolites^10^, metal–organic frameworks^11^ have been well explored for CO_2_ capture to meet some of the major objectives-high adsorption capacity, superior selectivity (CO_2_ over other gases such as N_2_), hydrophobicity, high attrition resistance, low-pressure drop, high mass transfer coefficient, and thermo-chemical stability and so on^12^. However, none of the adsorbents exhibits desired objectives during CO_2_ adsorption while dealing with mild CO_2_ content environment or at low partial CO_2_ pressure^12^. Although, it is well established that, synthetic zeolites (specifically cation exchanged zeolites e.g. NaA, NaK-A, 13X-Li) exhibit high CO_2_ adsorption capacity and superior CO_2_ over N_2_ selectivity while dealing with the flue gas (typically consists of 10–15% CO_2_), the presence of moisture dramatically reduces its performance^13,14^. In that sense, metal–organic frameworks (MOFs) have received significant attention for CO_2_ capture applications because of their remarkably high surface area, high adsorption capacity, enrich surface functionality and tunable pore size^15,16^.

The literature revealed that MIL-101(Cr) has been extensively explored for gas adsorption applications as it exhibited an appreciably high CO_2_ adsorption capacity of 40 mmol g^−1^ at 30 °C and 50 bar^17^. However, such a large adsorption capacity of MIL-101(Cr) can only be achieved at high CO_2_ adsorption pressure, and thus, MIL-101 lacks direct application in CO_2_ capture from flue gas without compression^18^. Although MIL-101 exhibited superior gas adsorption performance at high pressure, nearly 80% of its pore volume remains under-utilized for gas uptake at low pressure^19^. In this regard, various strategies such as ligand modification, amine impregnation, and incorporation guest material to the MIL-101(Cr) framework have been explored to enhance the CO_2_ adsorption properties by utilizing the unused pore volume^20^. Inclusion of guest material such as carbon nanotube, graphene oxide, metal ions, and amines into the MIL-101(Cr) framework significantly tune the pore size, pore volume, which allow CO_2_ to retain in the tunnels and cages by enhancing the interaction with pore wall. Advantageously, increase in microporosity in MOF augmented the CO_2_ adsorption potential which helps in the strong interaction of CO_2_ molecules with multiple adsorption sites of MOF^21^.

Amine groups introduced in the MIL-101(Cr) framework during pre or post synthesis can act as Lewis bases to strongly bind with CO_2_ molecules and increases the selectivity against other gases such as N_2_^21^. For instance, Chen et al*.* reported PEI-incorporated MIL-101(Cr) adsorbents exhibited ultra-high CO_2_ adsorption capacity (4.2 mmol g^−1^) at 0.15 bar and superior CO_2_/N_2_ selectivity (770) in the flue gas (0.15 bar CO_2_ and 0.75 bar N_2_) at 25 ºC^22^. However, the desorption of CO_2_ in amine-functionalized MIL-101(Cr) could be difficult due to their high heat of adsorption (even up to ~ 98 kJ mol^−1^)^23^. On the other hand, doping of metal ions in MIL-101(Cr) during solvothermal crystallization can be a better option to enhance CO_2_ adsorption property^24^. Zhou et al*.* doped Mg^2+^ in MIL-101(Cr) to achieve a 40% enhancement in CO_2_ uptake and ~ 4 times improvement in CO_2_/N_2_ selectivity compared to un-doped MIL-101(Cr) at 1 bar^24^. Similarly, composites of MIL-101(Cr) with carbon or silica-based material also exhibited enhanced CO_2_ adsorption capacity due to the improvement in the interaction of MOFs with CO_2_ molecules^25–28^. Chen et al*.* reported hybrid MIL-101(Cr)@MCM-41 composite which exhibited 79% enhancement in CO_2_ uptake capacity and 43% rise in CO_2_/N_2_ selectivity compared to the parent MIL-101(Cr), presumably due to the interaction between surface hydroxyl groups of MCM-41 and metal centers of MOF^25^. Moreover, Qasem et al*.* reported an enhancement of 35.9% in CO_2_ adsorption capacity at 24 °C and 1 bar pressure, after the incorporation of multiwall carbon nano tube (MWCNT) in MIL-101(Cr)^26^. In particular, synthetic carbon or/and silica-based MOFs have been developed to increase the CO_2_ adsorption capacity. However, direct utilization of agricultural waste material such as rice husk ash (RHA) containing both silica and carbon in MOF synthesis and its consequences on the CO_2_ adsorption properties are yet not extensively investigated. RHA has diversified applications ranging from pozzolanic material in the construction industry^29^ to feedstock for the development of various CO_2_ capture adsorbents^30^. RHA is amorphous/mesoporous in nature and has exposed silanol bonds which can enhance facile interactions with CO_2_ molecules. Therefore, it is of interest to study the CO_2_ adsorption performance such as working capacity, regenerability, CO_2_/N_2_ selectivity as well as isosteric heat of adsorption of RHA modified MIL-101(Cr) at low pressure (pertaining to flue gas condition) by interplaying with its structural properties.

Herein, we synthesized RHA incorporated MIL-101(Cr) using a varying amount of RHA in situ during the hydrothermal synthesis of MIL-101(Cr) and investigated the effect of RHA on the CO_2_ adsorption at 0 °C, 25 °C and 1 bar. Structural, morphological and chemical properties of RHA-MIL-101(Cr) are analyzed by powder X-ray diffraction (P-XRD), field emission scanning electron microscopy (FESEM), transmission electron microscopy (TEM), Fourier-transform infrared spectroscopy (FTIR), Raman spectroscopy, and energy dispersive spectroscopy (EDS). Textural properties (surface area, pore-volume, and pore size distribution) of RHA-MIL-101(Cr) are studied using N_2_ adsorption–desorption isotherm at − 196 °C and CO_2_ adsorption at 0 °C. The pure component adsorption isotherm of CO_2_ at 0 °C, 25 °C and N_2_ at 25 °C is investigated in the pressure range 0–1 bar. The observed adsorption behavior of the synthesized RHA incorporated MIL-101(Cr) is correlated with their textural properties. Further, adsorbents evaluation parameters such as working capacity, purity and CO_2_/N_2_ selectivity and adsorption thermodynamics were also evaluated. Our finding suggested that the studied RHA-MIL-101(Cr) represents a class of efficient CO_2_ adsorption material. Through this study, we also attempted to explore the potential utilization of the throw-away agriculture waste, RHA, for the synthesis of a value-added CO_2_ capture material.

## Results and discussion

**Scheme 1:**
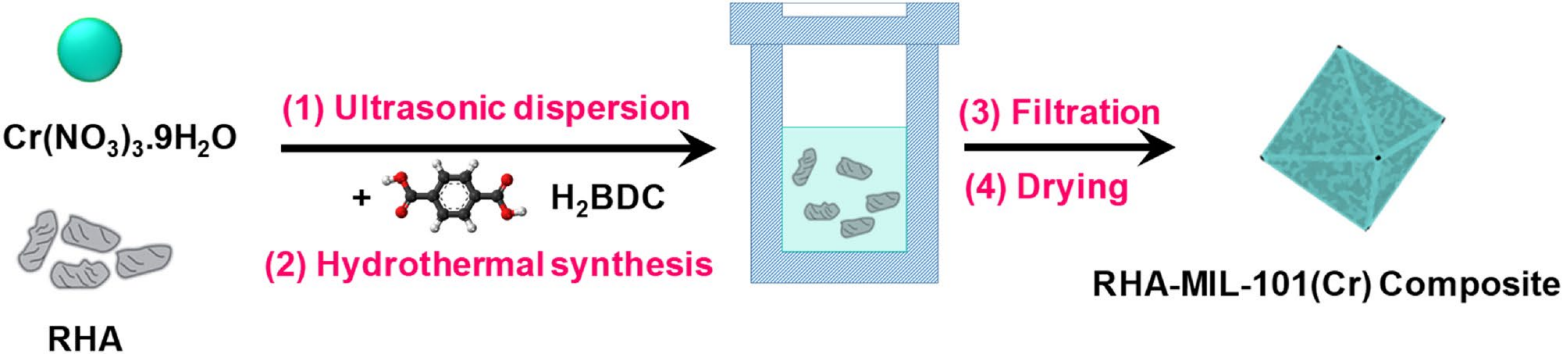
Schematic representation for the synthesis of RHA-MIL-101(Cr) composite.

RHA incorporated MIL-101(Cr) (RHA-MIL-101(Cr)-I and RHA-MIL-101(Cr)-II) are synthesized by incorporating pre-treated RHA in situ during the synthesis of MIL-101(Cr) under hydrothermal condition, as elaborated in the experimental section. The schematic representation of the RHA-MIL-101(Cr) composite synthesis is given in Scheme 1.

**Figure 1:**
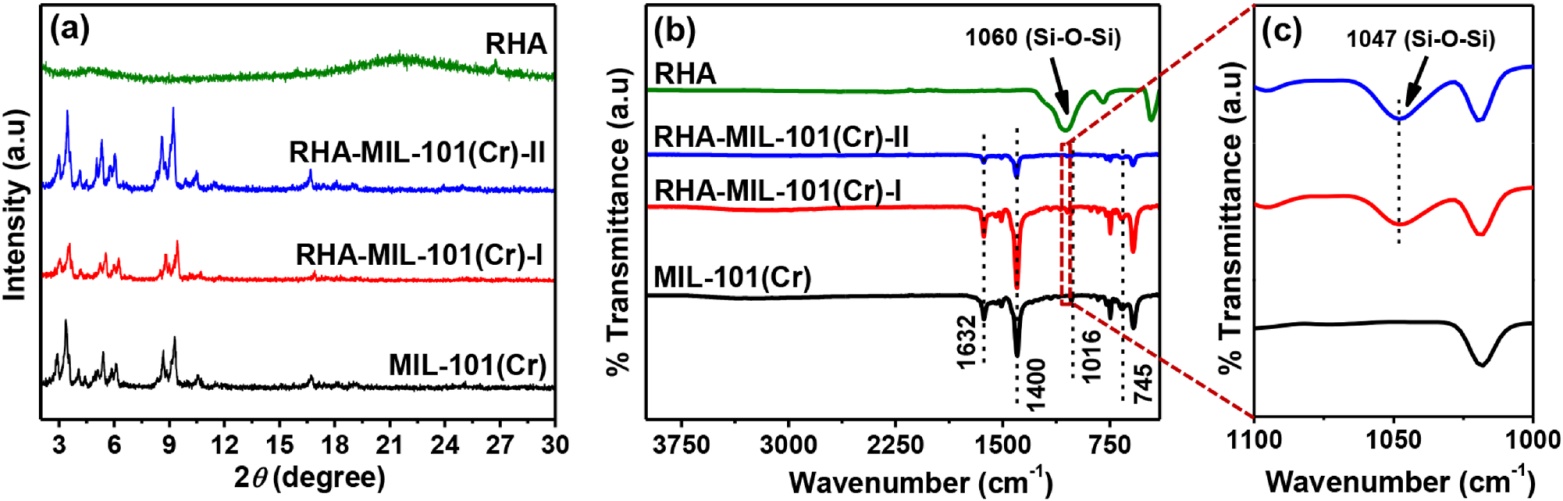
(**a**) P-XRD patterns, and (**b**,**c**) FTIR spectra of RHA, MIL-101(Cr), RHA-MIL-101(Cr)-I, and RHA-MIL-101(Cr)-II.

The P-XRD patterns of RHA, MIL-101(Cr), RHA-MIL-101(Cr)-I, and RHA-MIL-101(Cr)-II are shown in Fig. 1a, suggests that the crystalline framework of MIL-101(Cr) is well preserved even after the incorporation of RHA in MIL-101(Cr). The presence of characteristic peaks at 3.3°, 5.4°, 5.9°, 9.29°, and 16.71° for MIL-101(Cr) are in good agreement with previous reports^24,31^. It is also noted that in P-XRD patterns no new peaks from RHA are found, indicating the high dispersion and relatively low RHA content in the composites. Moreover, only a slight variation in the intensities of the peaks of the RHA-MIL-101(Cr) at lower 2*θ* is observed as compared to the pristine MIL-101(Cr), indicating no significant change in the crystallinity of framework upon incorporation of RHA in MIL-101(Cr) (Fig. S1)^25^.

The presence of a characteristic peak at 582 cm^−1^ corresponds to Cr–O vibration in the FTIR spectra (Fig. 1b) of MIL-101(Cr) and RHA-MIL-101(Cr) inferred the presence of MIL-101 framework^24^. A strong band appeared at 1400 cm^−1^ and 1632 cm^−1^ is assigned to the O–C–O symmetric stretching vibration of BDC linker present in MIL-101(Cr) framework^24,32^. Moreover, the incorporation of RHA in MIL-101 framework is further confirmed by the presence of signature bands at 1060 cm^−1^ and 1047 cm^−1^, corresponds to Si–O–Si vibration (Fig. 1c)^33^. Further, the presence of bands at 1016 cm^−1^ (assigned to in-plane bending vibration of C–H on the benzene ring), 884 cm^−1^ (assigned to out-of-plane bending vibration of C–H on the benzene ring) and 745 cm^−1^ (assigned to deviational vibration of carboxylate groups) are consistent with the literature reports^34,35^.

Raman Spectra of MIL-101(Cr), RHA-MIL-101(Cr)-I, and RHA-MIL-101(Cr)-II (Fig. S2) also exhibited signature bands at 1615, 1457, 1144, and 871 cm^−1^, confirming the presence of MIL-101(Cr) framework^36^. The characteristic peaks at 1457 and 1615 cm^−1^ are attributed to the respective symmetric and asymmetric vibrations of the carboxylate group of BDC linker of MIL-101(Cr), while, the peaks at 1144 cm^−1^ and 871 cm^−1^ are assigned to the vibration of the C–C bond between the benzene ring and the carboxylate group and the external plane deformation of the C–H link^37,38^.

Thermogravimetric analysis (TGA) is used to study the thermal stability of RHA-MIL-101(Cr) upon the incorporation of RHA in MIL-101(Cr) (Fig. S3). TGA traces of MIL-101(Cr), RHA-MIL-101(Cr) exhibit an initial weight loss in the temperature range of 100–160 °C due to the removal of guest water molecules from the large cage (d = 3.4 nm). The second weight loss in the range of 160–350 °C, can be ascribed to the loss of trapped water molecules from the relatively small cages (d = 2.9 nm)^35^. The weight loss beyond 350 °C is due to the decomposition of BDC and the structural framework of MIL-101(Cr)^20,39^. With an increase in carbon and silica content upon addition of RHA in MIL-101, the resulting composite RHA-MIL-101(Cr) demonstrating a superior hydrophobicity as well as thermal stability (Table S1).

**Figure 2:**
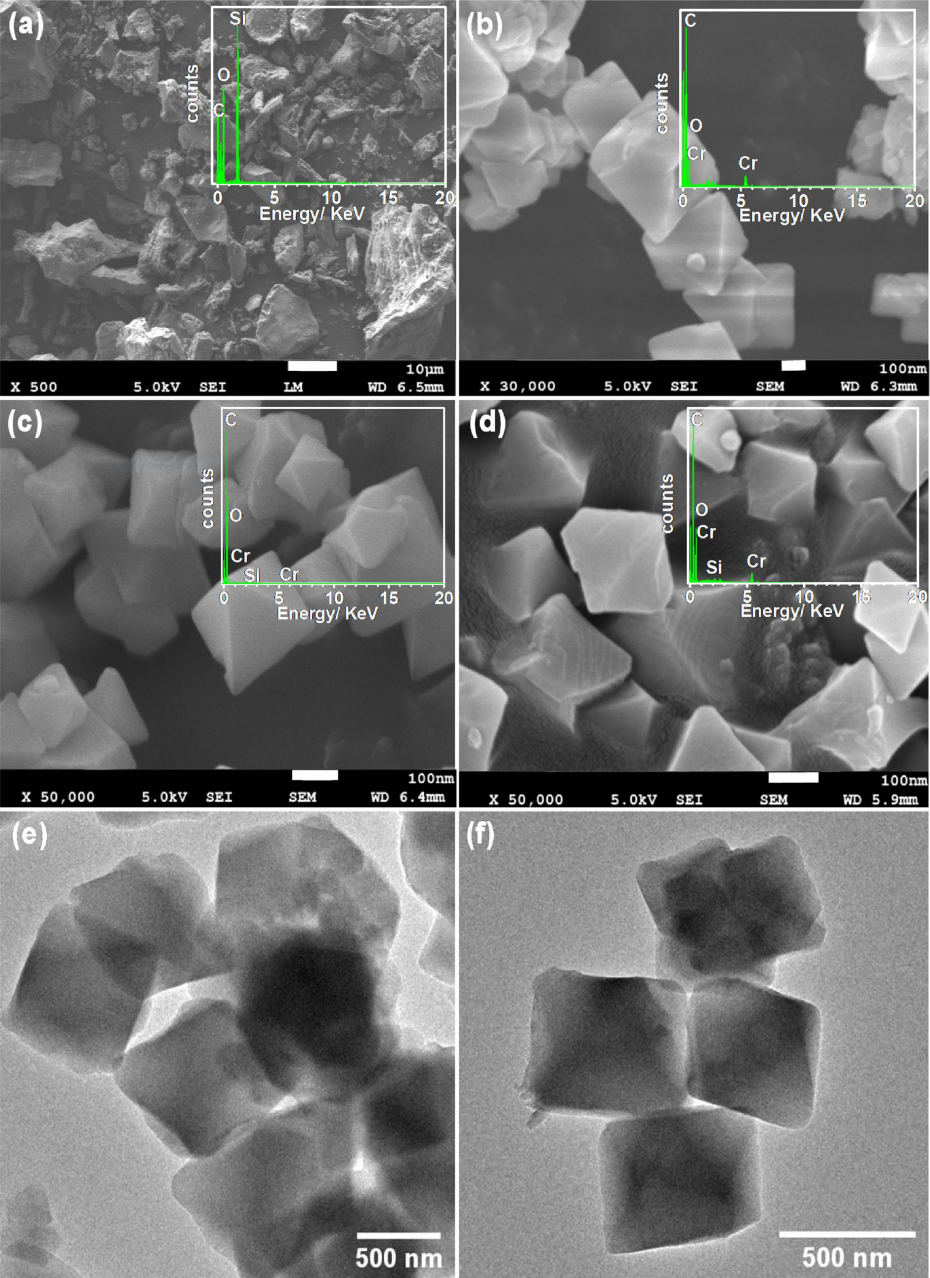
(**a**–**d**) SEM images (with corresponding EDS spectra in the inset) of (**a**) RHA, (**b**) MIL-101(Cr), (**c**) RHA-MIL-101(Cr)-I, (**d**) RHA-MIL-101(Cr)-II, (**e**,**f**) TEM images of (**e**) MIL-101(Cr) and (**f**) RHA-MIL-101(Cr)-II.

**Figure 3:**
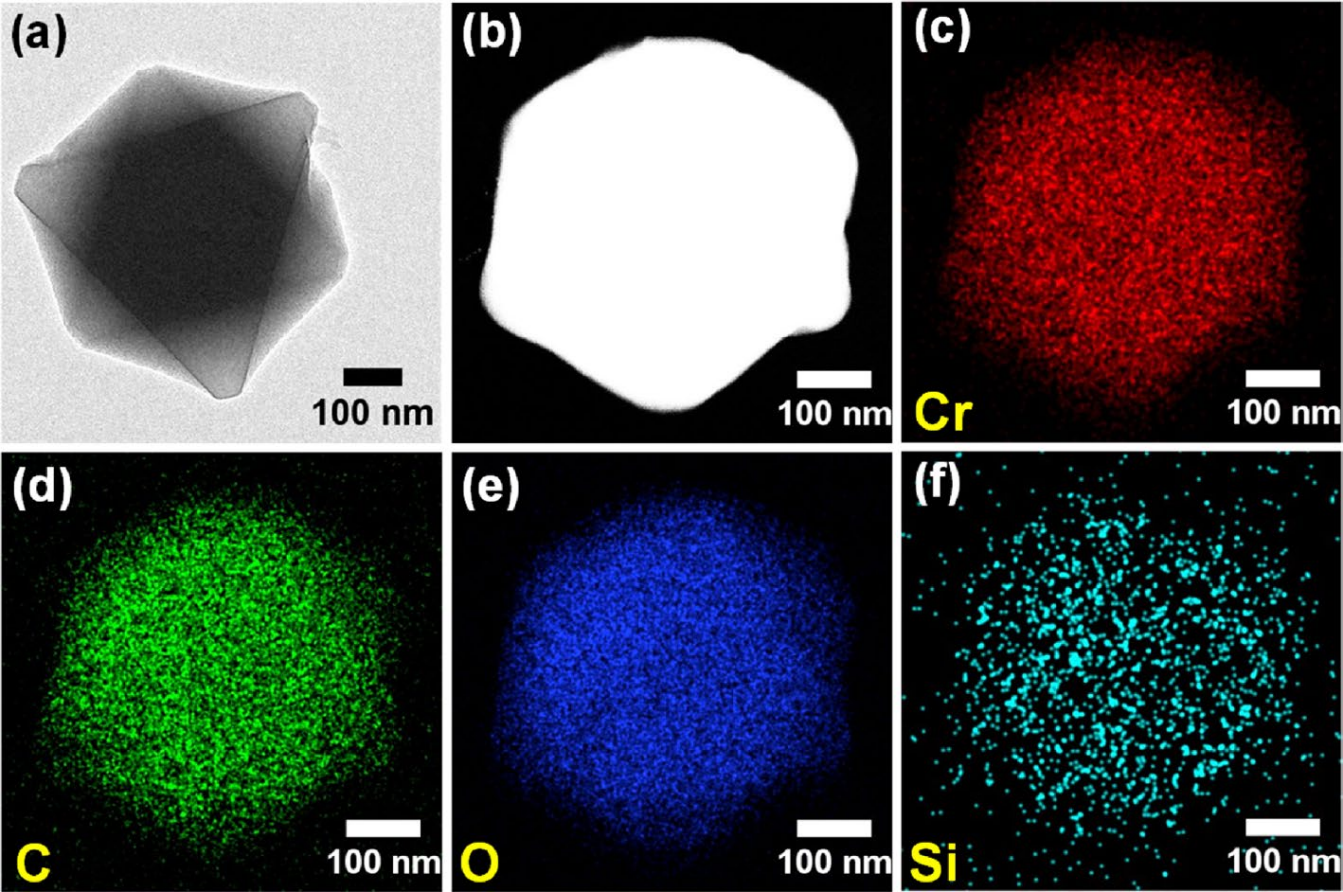
(**a**) TEM, (**b**) HAADF-STEM images and (**c**–**f**) the corresponding EDS elemental mapping of RHA-MIL-101(Cr)-II.

Elemental analysis shows 13–23 wt% higher carbon content in RHA-MIL-101(Cr) as compared to MIL-101(Cr). The increasing thermal stability upon rise in carbon and silica content in composite MOFs is in good agreement with the previous reports^35,40^*.* Consistent with P-XRD, FTIR, and Raman spectra, the SEM and TEM results also suggest the intactness of structural and morphological integrity of MIL-101(Cr) even upon the incorporation of RHA (Fig. 2 and Fig. S4). TEM images of RHA-MIL-101(Cr) displays the presence of particles with size in the range of 100–400 nm having octahedron morphology analogous to MIL-101(Cr) (Fig. 2 and Fig. S5). This is presumably due to the complete dispersion of RHA (0.31–0.62 wt% to the metal content of MIL-101) into the MIL-101(Cr) framework. Moreover, EDS spectra of RHA-MIL-101(Cr)-I and RHA-MIL-101(Cr)-II exhibited the presence of both Cr and Si (Fig. 2). Further, TEM-EDS elemental mapping of a single particle of RHA incorporated MIL-101 infers the presence of silica spread all over the octahedron particle, evidence the complete dispersion of RHA incorporated in MIL-101(Cr) framework (Fig. 3).

**Figure 4:**
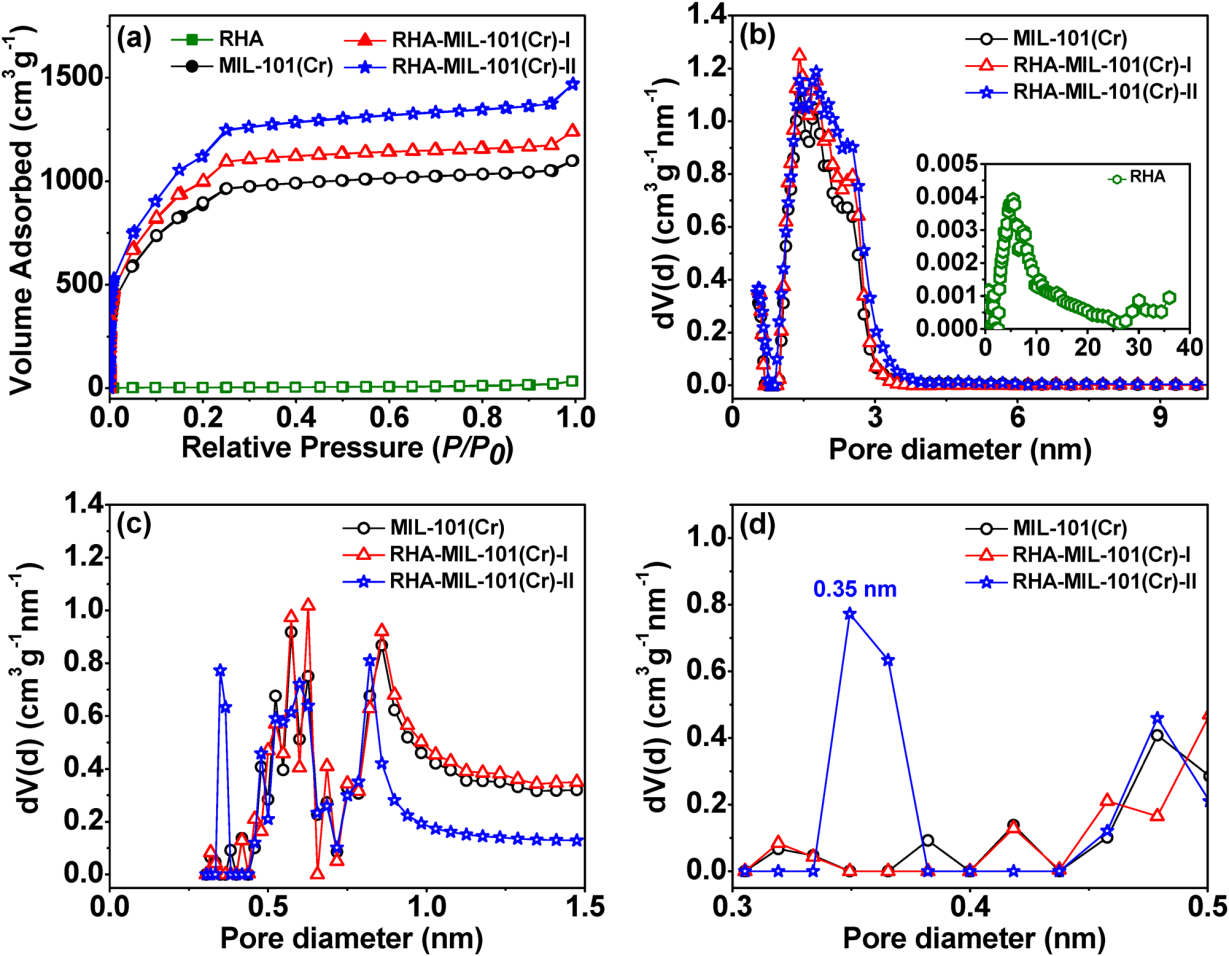
(**a**) N_2_ adsorption–desorption isotherms at − 196 °C, and (**b**) pore size distribution of RHA, MIL-101(Cr), RHA-MIL-101(Cr)-I, and RHA-MIL-101(Cr)-II obtained from N_2_ adsorption isotherm. (**c**,**d**) Micropore size distribution of MIL-101(Cr), RHA-MIL-101(Cr)-I, and RHA-MIL-101(Cr)-II obtained from CO_2_ adsorption isotherm at 0 °C.

To investigate the possible structural changes occurred due to incorporation of RHA in the MIL-101(Cr) framework, the textural properties (BET surface area, total pore volume, and micropore volumes) of MIL-101(Cr), RHA-MIL-101(Cr)-I and RHA-MIL-101(Cr)-II are studied and compared with the parent MIL-101(Cr) (Fig. 4a). Table 1 summarizes all the textural data of RHA-MIL-101(Cr)-I and RHA-MIL-101(Cr)-II along with MIL-101(Cr) and RHA. Notably, N_2_ sorption isotherm at − 196 °C of MIL-101(Cr), RHA-MIL-101(Cr)-I and RHA-MIL-101(Cr)-II exhibited a type I isotherm with a secondary uptake in the pressure range of 0.15–0.25 bar, suggesting the presence of two different microporous windows, and is consistent with MIL-101(Cr) framework^24^. On the other hand, type III isotherm of RHA is consistent with the mesopores or amorphous nature of RHA^32^. Notably, RHA-MIL-101(Cr)-I and RHA-MIL-101(Cr)-II exhibited an increment of 12.6% and 27.6% in their specific surface area, respectively compared to MIL-101(Cr). Such surface area enhancement is often seen in MOF composite by the inclusion of guest material during solvothermal crystallization, which is well documented in literature^24,25,40,41^. Moreover, the total pore volume (at *P/P*_*0*_ = 0.99) increased by 33%, from 1.70 cm^3^ g^−1^ (for MIL-101(Cr)) to 2.27 cm^3^ g^−1^ (RHA-MIL-101(Cr)-II). In addition, the micropore volume (from t-plot) also increased from 1.33 cm^3^ g^−1^ (for MIL-101(Cr)) to 1.57 cm^3^ g^−1^ (RHA-MIL-101(Cr)-I) and 1.73 cm^3^ g^−1^ (RHA-MIL-101(Cr)-II). The observed enhancement in specific surface area and micropore volume of RHA-MIL-101(Cr) composites can be attributed to coordination effect between the surface silanol group of RHA and the metal centers of MIL-101(Cr)^40,41^.

**Table 1:** Textural properties and CO_2_ and N_2_ adsorption capacity of MIL-101(Cr), RHA-MIL-101(Cr)-I, and RHA-MIL-101(Cr)-II.

Materials	S_BET_ (m^2^ g^−1^)	Pore volume			CO_2_ uptake (mmol g^−1^)			N_2_ uptake (mmol g^−1^)	
		V_total_ (cm^3^ g^−1^)	V_micro_ (cm^3^ g^−1^)	V_ultramicro_ (cm^3^ g^−1^)	0 °C	25 °C		25 °C	
					1 bar	0.15 bar	1 bar	0.75 bar	1 bar
MIL-101(Cr)	3325	1.70	1.33	0.120	3.60	0.36	2.20	0.24	0.33
RHA-MIL-101(Cr)-I	3744	1.91	1.57	0.123	3.70	0.43	2.79	0.27	0.36
RHA-MIL-101(Cr)-II	4249	2.27	1.73	0.133	3.54	0.70	2.51	0.20	0.27
RHA	14	0.05	NC	NC	0.13	0.01	0.08	NC	NC

The NLDFT pore size distribution for MIL-101(Cr), RHA-MIL-101(Cr)-I, and RHA-MIL-101(Cr)-II obtained from the N_2_ adsorptions at − 196 °C and CO_2_ adsorption isotherm at 0 °C are shown in Fig. 4b–d, respectively. Figure 4b displayed analogous pore size distribution for MIL-101(Cr) and RHA-MIL-101(Cr) suggest no significant change in the pore size distribution of MIL-101(Cr) upon RHA incorporation (Fig. 4b). Notably, for accessing the ultra-micropores (pores < 0.7 nm), CO_2_ adsorption at 0 °C is preferred over N_2_ at − 196 °C due to the higher saturation pressure (34.85 bar) and rapid diffusion rate of CO_2_ over N_2_^42^. Hence, the micropore size distribution of RHA-MIL-101(Cr)-II inferred the formation of new ultra-micropore in the range of 0.35 nm which is consistent with the observed increment in cumulative micropore volume for RHA-MIL-101(Cr)-II as compared to MIL-101(Cr) and RHA-MIL-101(Cr)-I (Fig. 4d, and Figs. S6–S8). It is worth noticing that, the ultra-microporosity also increased upon RHA loading in MIL-101(Cr) framework, which indicate the synergetic combination of two components. Previous reports also evidence that fine-tuning of pores in MOF may occur due to the interaction of silanol bonds with metal centres^40,41^.

**Figure 5:**
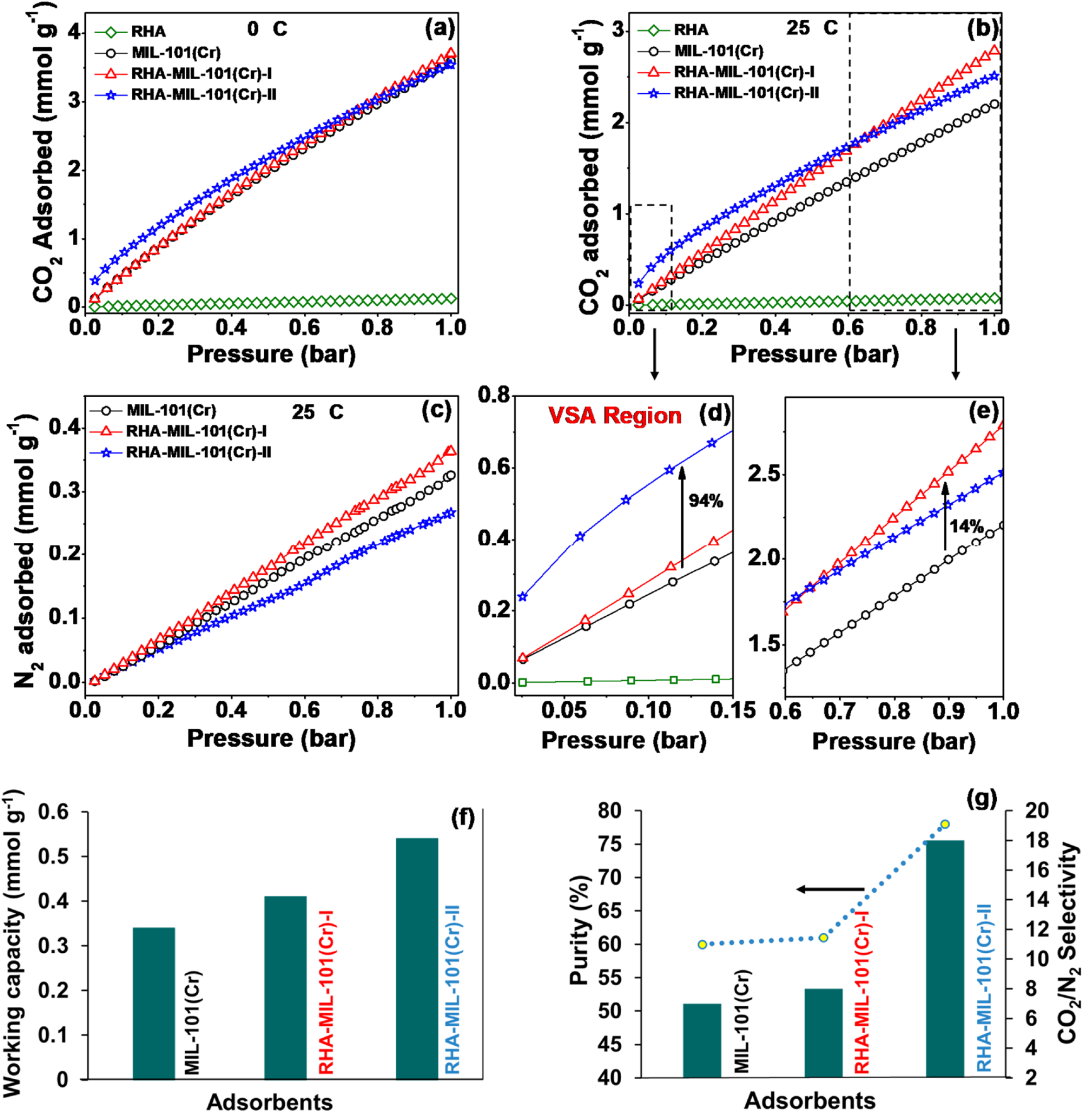
(**a**) CO_2_ adsorption isotherms at 0 °C, (**b**) CO_2_ adsorption isotherms at 25 °C, (**c**) N_2_ adsorption isotherms at 25 °C in the pressure range of 0–1 bar, (**d**,**e**) CO_2_ adsorption isotherms in the pressure ranges of 0–0.15 bar (**d**) and 0.6–1 bar (**e**) at 25 °C, (**f**) Regenerability, working capacity at 25 °C, and (**g**) CO_2_/N_2_ adsorption selectivity, purity at 25 °C (as per flue gas condition).

The CO_2_ and N_2_ adsorption capacity of MIL-101(Cr) and RHA-MIL-101(Cr) composite in the pressure range of 0–1 bar at 25 ºC are shown in Fig. 5a–c, respectively. In accordance with the enhanced porosity characteristics of RHA-MIL-101(Cr), both RHA-MIL-101(Cr)-I and RHA-MIL-101(Cr)-II exhibited high CO_2_ adsorption capacity compared to MIL-101(Cr) (Fig. 5a,b,d,e). The observed enhancement can be attributed to the improved textural properties and availability of silanol functionalities in the pores of RHA-MIL-101(Cr) due to the incorporation of RHA in MIL-101(Cr) framework^40^. The loading content of RHA in MIL-101(Cr) tuned the CO_2_ adsorption performance of RHA-MIL-101(Cr). The result suggests that the incorporation of even small amount of RHA into MIL-101(Cr) framework facilitates the formations of additional micropores those provide extra CO_2_ adsorption binding site in RHA-MIL-101(Cr). Among the synthesized RHA incorporated MIL-101(Cr), RHA-MIL-101(Cr)-I exhibits the highest CO_2_ uptake 2.79 mmol g^−1^ (12.27 wt %) at 25 °C and 1 bar, which is 27% higher than of MIL-101(Cr). Moreover, RHA-MIL-101(Cr)-II also exhibited 14% higher CO_2_ uptake (2.51 mmol g^−1^) at 25 °C than MIL-101(Cr) (2.20 mmol g^−1^). RHA-MIL-101(Cr)-I also showed higher CO_2_ uptake (3.70 mmol g^−1^) at 0 °C, compared to RHA-MIL-101(Cr)-II and MIL-101(Cr). The BET surface area and pore volume are ordered as RHA-MIL-101(Cr)-II > RHA-MIL-101(Cr)-I > MIL-101(Cr). The observed enhanced CO_2_ adsorption capacity for RHA incorporated MIL-101 is consistent with the enhancement of 12–27% in BET surface area, 12–33% in total pore volume and 18–30% in micropore volume as compared to the parent MIL-101(Cr) (Fig. 4d, Table 1). It is noteworthy that, due to inadequate pore filling, RHA-MIL-101(Cr)-II exhibits comparatively less CO_2_ adsorption capacity than RHA-MIL-101(Cr)-I at 1 bar, although it has higher BET surface area and pore volume^43^. Evidently, higher BET surface area and pore volume may have correlated positively with high pressure CO_2_ adsorption, but the presence of micropore in the adsorbent plays a major role in enhancing CO_2_ interaction at low pressure^43,44^. In support of the aforementioned statement, RHA-MIL-101(Cr)-II exhibited the finest increase in CO_2_ adsorption capacity at 0.15 bar (94%) as compared to RHA-MIL-101(Cr)-I (Fig. 5d), due to presence of excess micropore volume which may have direct relevance for CO_2_ capture under flue gas condition^40,44^. As seen in Fig. S9, there is a linear trend between the CO_2_ adsorbed with increase in ultra-micropore volume among all the studied adsorbents, which indicates the presence of ultra-micropore smaller than 0.7 nm is greatly responsible for enriched CO_2_ adsorption potential at low pressure^44^. Further, a good agreement of the Sips fitting parameters with the experimental data (R^2^ ∼ 0.99) suggest the efficient interaction of CO_2_ molecules with the pore wall of RHA-MIL-101(Cr), due to the heterogeneity in the adsorbent surface (Fig. S10, Table S2)^45,46^. Concurrently, the adsorption of N_2_ is found to be least in the case of RHA-MIL-101(Cr)-II as compared to RHA-MIL-101(Cr)-I and MIL-101(Cr) (Fig. 5c) presumably due to the poor diffusion of N_2_ into the ultra-micropores having pore diameter less than the kinetic diameter (0.36 nm) of N_2_^47^.

Apart from high CO_2_ adsorption capacity, RHA-MIL-101 (Cr) also exhibits low isosteric heat of adsorption, lying in the narrow range of weak physisorption, indicating that adsorbents need less energy during regeneration. As depicted in Fig. S11, the derived Q_st_ value for MIL-101(Cr) (19 kJ mol^−1^) at low surface coverage is quite accordance with the existing literature^48,49^. Upon RHA incorporation, the Q_st_ value was increased slightly for RHA-MIL-101(Cr)-I (20 kJ mol^−1^) and RHA-MIL-101(Cr)-II (30 kJ mol^−1^) at low surface coverage, attributed to the strong electrostatic interaction for CO_2_ with narrow pore size distribution^49^. Notably, the Q_st_ value of RHA-MIL-101(Cr) is at par with the several established MOF based composites available in the literature (Table S3).

Separation of CO_2_ from flue gas using vacuum swing adsorption (VSA) technique is considered to be one of the most energy-efficient and economical processes with lower regeneration time^50^. In addition to the high CO_2_ adsorption capacity, adsorbents should possess essential requirements such as high CO_2_ working capacity, superior selectivity for CO_2_ over N_2_, and high CO_2_ purity for post-combustion CO_2_ capture^51^. Therefore, the performance of the synthesized RHA-MIL-101(Cr) adsorbents is evaluated for the separation of CO_2_ from industrial flue gas using VSA technique, based on the data obtained from single gas adsorption isotherm at 0.15 bar of CO_2_ and 0.75 bar of N_2_. The CO_2_ separation performance of RHA incorporated MIL-101(Cr) along with the parent MIL-101(Cr) at 25 °C is shown in Fig. 5f,g and listed in Table S4. RHA-MIL-101(Cr)-II exhibits the best performance with highest working capacity (0.54 mmol g^−1^), and high purity (78%) as compared to RHA-MIL-101(Cr)-I and MIL-101(Cr). The observed high CO_2_ working capacity for RHA-MIL-101(Cr)-II, suggesting that there would be a significant reduction in the adsorbent replacement time and capital cost associated with the adsorbent amount for CO_2_ adsorption application^52^. Further, the CO_2_/N_2_ selectivity of 18 observed for RHA-MIL-101(Cr)-II is significantly higher as compared to MIL-101(Cr) (CO_2_/N_2_ selectivity = 7). The observed more than two-fold enhancement in CO_2_/N_2_ selectivity for RHA-MIL-101(Cr)-II could be a consequence of the exceptionally larger polarizability and quadrupole moment of CO_2_ (29.11 × 10^–25^ cm^−3^ and 4.30 × 10^−26^ esu^−1^ cm^−1^, respectively) than that of N_2_ (17.40 × 10^−25^ cm^−3^ and 1.52 × 10^−26^ esu^−1^ cm^−1^, respectively) and the steric effect of adsorbing molecules (CO_2_, N_2_) on the adsorbent surface^45^. Notably, micropore size distribution obtained from CO_2_ adsorption isotherm at 0 °C, also inferred the generation of new ultra-micropores (pore diameter of 0.35 nm) in RHA-MIL-101(Cr)-II (Fig. 5c), which can significantly tune the preferential adsorption of CO_2_ over N_2_ (kinetic diameter: CO_2_ = 3.3 nm and N_2_ = 3.6 nm) and hence resulted in superior CO_2_/N_2_ selectivity for RHA-MIL-101(Cr)-II. Besides, owing to high CO_2_/N_2_ selectivity, RHA-MIL-101(Cr)-II can also recover highly pure CO_2_ from the flue gas. Therefore, RHA-MIL-101(Cr)-II exhibited promising characteristics for the separation/purification of CO_2_/N_2_ mixture, even under ambient conditions.

The microporous analysis revealed that the incorporation of RHA in the MIL-101(Cr) framework significantly increased the specific surface area, micropore volume and also tuned the pore diameter, which resulted in a remarkable improvement in CO_2_ adsorption properties for RHA-MIL-101(Cr). The CO_2_ adsorption capacity of RHA-MIL-101(Cr)-I at 1 bar and 25 °C is at par or even higher than some of the popular MOFs based composite (using carbon or silica), which are being widely investigated as significant materials for CO_2_ capture (Table S3). The observed higher CO_2_ adsorption behavior of RHA-MIL-101(Cr) at low pressure (0.15 bar) is well in accordance with the microporous nature of these adsorbents^25,40^. The synergetic interaction between the silanol groups of RHA and the metal sites of MIL-101(Cr) induced structural changes which is likely to be responsible for the generation of microporosity in RHA-MIL-101(Cr). Literature also revealed that, the incorporation of heterogeneous material such as silica in MOF framework may acts as structure directing agent to modulate the textural characteristics of MOF crystal, and consequently influence the CO_2_ adsorption performance of MOF^40,41^. For instance, Chen et al*.* reported 15.9 and 39% enhancement in CO_2_ uptake and kinetics at 1 bar and 25 °C over HKUST-1@SBA-15 composite, respectively, where the micropores present in composite played a key role in increasing CO_2_ adsorption capacity and the mesoporosity available in SBA-15 enhanced the CO_2_ adsorption kinetics behavior^40^. Therefore, the tuned CO_2_ adsorption behavior of RHA incorporated MIL-101(Cr) is of particular importance for utilizing RHA-MIL-101(Cr) for both flue gas separation and bulk CO_2_ gas purification applications.

## Conclusions

This work demonstrates high CO_2_ adsorption performance achieved by the incorporation of rice husk ash (RHA), a waste material, in situ during the synthesis of MIL-101(Cr) under hydrothermal condition. RHA incorporated MIL-101(Cr) exhibited high specific surface area, high micropore volume and tuned pore diameter as compared to the parent MIL-101(Cr). Moreover, incorporation of silica-rich RHA fine-tuned the interaction of CO_2_ molecules with pore walls due to the presence of silanol bonds and enhanced the utilization of large pore volume. Consequently, the RHA incorporated MIL-101(Cr) exhibited 27% higher CO_2_ adsorption capacity compared to MIL-101(Cr) at 25 °C, attributed to the enhancement in total pore volume and micropore volume by 33% and 30%, respectively compared to MIL-101(Cr). It is worth noticing that, RHA-MIL-101(Cr)-II displayed better CO_2_ uptake at low pressure (0.15 bar) as compared to RHA-MIL-101(Cr)-I due to the generation of ultra-micropore in the range of 0.35 nm. RHA-MIL-101(Cr)-II also possesses high working capacity (0.54 mmol g^−1^), high purity (78%) and superior CO_2_/N_2_ selectivity (18) compared to RHA-MIL-101(Cr)-I and MIL-101(Cr) under vacuum swing based adsorption at flue gas condition (0.15 bar CO_2_ vs. 0.75 bar N_2_). Hence, incorporation of agriculture waste RHA in MIL-101(Cr) provided an environmentally benign route to fine-tune the textural and porous characteristics of MIL-101(Cr) to achieve enhanced CO_2_ adsorption capacity. Further investigations are being carried out in the laboratory to evaluate the behavior of RHA-MIL-101(Cr) for high-pressure CO_2_ adsorption. Taking into account of superior CO_2_ adsorption performance, RHA incorporated MIL-101(Cr) could be a potential adsorbent for purification and separation of gases for industrial application.

## Experimental

### Materials and characterization

Terephthalic acid (H_2_BDC, 98%) was purchased from Thomas Baker. Chromium (III) nitrate nonahydrate (Cr(NO_3_)_3_·9H_2_O, 98%) was obtained from Sigma Aldrich. Nitric acid (68–70%), acetone (≥ 99%), hydrochloric acid (36.5–38%) and distilled water were provided by Merck. Ethanol (99.9%) was purchased from SD Fine Chemical. Rice husk ash (RHA) was provided by Nishant Enterprises India and pre-treated before use. All these materials and reagents were utilized without any further purification. Ultra-high purity (99.999%) grade helium, carbon dioxide, and nitrogen were purchased from Inox air product ltd, India for low-pressure adsorption measurement.

Powder X-ray diffraction (P-XRD) patterns of the adsorbents were obtained with a Rigaku Smart Lab automated powder X-ray diffractometer (λ = 0.154 nm) at a step size of 0.01° over a 2*θ* range from 2 to 80°. Fourier transform infrared spectroscopy (FTIR) analysis was carried out to probe the vibrational properties of the chemical functional groups present in the studied adsorbents. The spectra were recorded using a spectrometer equipped with an attenuated total reflectance (FTIR/ATR 229 model FTIR-STD-10, PerkinElmer, MA, U.S.A) in the wavenumber range 4000–500 cm^−1^. To analyze the thermal stability and dehydration characteristics of the studied adsorbents, thermogravimetric study (TGA) was performed using a Shimadzu TGA-50 Series thermal analyzer at a heating rate of 5 °C min^−1^ from 25 to 800 °C under N_2_ atmosphere. Prior to FTIR and TGA experiment, all the samples were dried at 110 ºC and allowed to cool at room temperature and then stored in a desiccator until the testing began. Carbon content in the sample was determined using Leco CS 230 Carbon/Sulfur analyzer. Field emission scanning electron microscopic (FESEM) images and EDS spectra were recorded on a Carl Zeiss Supra-55 and EDS Oxford instruments (X-Max, energy-dispersive X-ray spectrometer) respectively. Similarly, transmission electron microscopic (TEM) images and EDS elemental mapping were acquired from FEI Talos 200S transmission electron microscope equipped with 200 kV Field Emission Gun (FEG). The samples were well dispersed in ethanol by sonication and drop casted onto a copper supported carbon film. Raman spectra were collected using Labram HR evolution Raman spectrometer (Horiba Jobin Yvon) equipped with an argon-ion laser (λ = 532 nm). Sub-critical Nitrogen sorption isotherm and textural properties (specific surface area, pore-volume, pore size distribution) were measured at − 196 °C using a Quantachrome Autosorb iQ_2_ TPX automated gas sorption system. Brunauer–Emmett–Teller (BET) equation was used to calculate the specific surface areas and applied to the adsorption data over the relative pressure (*P/P*_*0*_) range of 0.05–0.20. The total pore volume (V_total_) was calculated from the amount of adsorbed N_2_ at *P/P*_*0*_ = 0.99 using the single point adsorption method. Micropore volume (V_micro_) calculated by the t-plot method and ultra-micropore volume (V_ultramicro_) for the pores < 0.7 nm was calculated by NLDFT method from CO_2_ adsorption isotherm at 0 °C (assuming carbon adsorbent having slit pores kernel). The above textural properties are evaluated using Quantachrome ASiQwin data processing software equipped along with the instrument. Before gas sorption measurement, all the samples were outgassed at 160 °C for 15 h under ultra-high vacuum (10^–6^ mbar).

### Synthesis of MIL-101(Cr)

MIL-101(Cr) was synthesized under hydrothermal condition following our previously reported method^32^. Briefly, Cr(NO_3_)_3_·9H_2_O (2.0 g), H_2_BDC (0.833 g), HCl (0.416 mL), and H_2_O (30 mL) were mixed under sonication (30 min) at room temperature. The resulting mixture was transferred to a 50 mL Teflon-lined autoclave and heated at 220 °C for 8 h in a programmable oven. After the completion of the reaction, the autoclave was allowed to cool down to the ambient temperature, and the obtained green solid was separated from the solution by centrifugation. Subsequently, the solid was washed twice with hot distilled water, acetone and hot ethanol. Further, the green solid was suspended in the ethanol–water mixture (30 mL, 95/5 v/v) and heated at 80 °C for 8 h in a Teflon-lined autoclave. Finally, the obtained green solid was dried at 80 °C for 12 h under vacuum.

### Synthesis of RHA-MIL-101(Cr)

Prior to the synthesis of RHA-MIL-101(Cr), the supplied RHA was pre-treated. Initially, the supplied RHA was grounded with the help of mortar pastel for 60 min and subsequently sieved by 200-micron mesh sieves to obtain uniform particle size. The resultant powdered RHA was purified by the treatment of (1:1, v/v) HNO_3_/distilled water and finally dried at 120 °C for 12 h under vacuum. RHA-MIL-101(Cr) was synthesized using the procedure analogous to that of MIL-101(Cr), where a specified amount of RHA was suspended in the reaction mixture containing the ingredient required for the synthesis of MIL-101(Cr). RHA-MIL-101(Cr)-I and RHA-MIL-101(Cr)-II were obtained by incorporating 6.25 mg and 12.5 mg of RHA, respectively during the synthesis. The obtained RHA-MIL-101(Cr) was purified and activated under similar condition used for MIL-101(Cr).

### CO_2_ and N_2_ adsorption measurements

Pure component CO_2_ and N_2_ sorption isotherms of MIL-101(Cr), RHA-MIL-101(Cr)-I and RHA-MIL-101(Cr)-II were obtained using Quantachrome Autosorb iQ_2_ TPX automated gas sorption system at varied temperature (0 °C and 25 °C) for CO_2_ and at 25 °C for N_2_ in the pressure range of 0–1 bar by using a static volumetric technique. A thermostatic bath was used to control the adsorbent temperature with a precision of ± 0.01 °C. Prior to the adsorption experiments, approximately 0.1 g of sample was outgassed at 160 °C for 15 h by using a turbo-molecular vacuum pump. A low heating rate (3 °C min^−1^) was chosen to allow steady removal of moisture from the sample by avoiding any structural changes of the adsorbents.

### Adsorption isotherm modelling

To evaluate the adsorption affinity between the adsorbate and the adsorbent over the pressure range (0–1 bar), the CO_2_ and N_2_ adsorption data were modelled by fitting them to the non-linear form of Sips equation respectively which are expressed as below.

1 $$q= \frac{{q}_{s}{({b}_{s}p)}^{1/s}}{1+{({b}_{s}p)}^{1/s}}$$

where p is the equilibrium adsorbate pressure (bar), q is the adsorption capacity (mmol g^−1^), q_s_ is the saturated adsorption capacity (mmol g^−1^), and b_s_ is the affinity constant for Sips model. The parameter s is usually less than unity and characterizes the heterogeneity of the adsorption system^53^. The values of parameters of the Sips model can be evaluated by non-linear curve fitting of the respective isotherm data.

### Adsorption thermodynamics

The thermodynamic property such as the isosteric heat of adsorption at a given CO_2_ adsorption capacity (q) was calculated from the isotherm data at two different temperatures (0 and 25 °C) by applying the Clausius–Clapeyron equation, as represented in Eq. (2).

2 $${\left(\frac{\partial (\mathit{ln}P)}{\partial \left(1/T\right)}\right)}_{q}= \frac{{Q}_{st}}{R}$$

where Q_st_ and R are the isosteric heat of adsorption and the universal gas constant, respectively. The isosteric heat of adsorption using Eq. (2) can also be obtained directly from Quantachrome ASiQwin data processing software equipped along with the instrument.

### Adsorbent evaluation parameters for CO_2_ capture

Performance of the synthesized adsorbents is evaluated for their possible application for CO_2_ adsorption from low CO_2_ content environment such as flue gas emitted from thermal power plants having a lower partial pressure of CO_2_ than N_2_. In this context, CO_2_/N_2_ selectivity, working capacity of CO_2_, regenerability of CO_2_ and purity of CO_2_ captured of the synthesized adsorbents were investigated by retrieving the data from their pure component adsorption isotherm at 0.15 bar of CO_2_ and 0.75 bar of N_2_. Practically vacuum swing adsorption (VSA) techniques could be more suitable to capture CO_2_ from flue gas to avoid the cost associated with compression or pressurization of CO_2_ during adsorption. In VSA, adsorption occurs at atmospheric pressure, and desorption occurs at sub-atmospheric pressure. Thus, by following Bae-Snurr criteria, a pressure range of 0–1 bar is often used to provide sufficient information about the evaluation parameter of the adsorbents under ambient conditions theoretically (without doing the actual VSA experiment in packed bed reactor)^51,54^.

- CO_2_ working capacity (WC) is the difference in CO_2_ adsorption capacity between adsorption and regeneration conditions = q_CO2_^ads^ − q_CO2_^des^
- Regenerability (R) = (WC/q_CO2_^ads^) × 100 (%)
- CO_2_ over N_2_ selectivity (S_CO2/N2_) = (q_CO2_^ads^/q_N2_^ads^)/(p_CO2_^ads^/p_N2_^ads^)
- Purity of CO_2_ = q_CO2_^ads^/(q_CO2_^ads^ + q_N2_^ads^) × 100 (%)

where q_CO2_^ads^ and q_N2_^ads^ are the amounts of CO_2,_ and N_2_ adsorbed at their respective equilibrium partial pressures (p_CO2_ and p_N2_). q_CO2_^des^ is the amounts of CO_2_ adsorbed at its desorption pressure. In general, for coal-fired power plants, the flue gas was generated at a total pressure of approximately 1 bar having a CO_2_ concentration of 15% and an N_2_ concentration of 75%^55^. Under these conditions, the corresponding partial pressure was 0.15 bar for CO_2_ and 0.75 bar for N_2_. For the VSA process, the CO_2_ partial pressure in the adsorption region is 0.15 bar and for desorption is 0.01 bar was considered for this evaluation.

## Supplementary information

Supplementary Information.
