# Supplementary material for: In situ casting of rice husk ash in metal organic frameworks induces enhanced CO2 capture performance
Source: Sci Rep. 2020 Nov 19;10:20219. doi: 10.1038/s41598-020-77213-9 (PMC7678836; doi:10.1038/s41598-020-77213-9)
Supplement: Supplementary file 1 — Supplementary Information. [file 41598_2020_77213_MOESM1_ESM.pdf]

Supplementary Information

In situ casting of rice husk ash in metal organic frameworks induces enhanced CO_2 capture performance

Debashis Panda,[1] Chanchal Saini,[2] E. Anil Kumar,[3] and Sanjay Kumar Singh[*2]

[1]Discipline of Mechanical Engineering, [2]Discipline of Chemistry, Indian Institute of Technology Indore, Simrol, Indore 453552, India

[3]Department of Mechanical Engineering, Indian Institute of Technology Tirupati, Tirupati 517506, India

E-mail address: sksingh@iiti.ac.in (SKS)

……………………………………………………………………………………………………

Table of Contents

Crystallinity calculation from the P-XRD pattern of (a) MIL-101(Cr), (b) RHA-MIL-101(Cr)-I, and (c) RHA-MIL-101(Cr)-II	**Figure S1**
Raman spectra of RHA, MIL-101(Cr), RHA-MIL-101(Cr)-I, and RHA-MIL-101(Cr)-II	**Figure S2**
TGA curves of (a) MIL-101(Cr), RHA-MIL-101(Cr)-I, RHA-MIL-101(Cr)-II and (b) RHA	**Figure S3**
FESEM image of (a) RHA, (b) MIL-101(Cr), (c) RHA-MIL-101(Cr)-I, and (d) RHA-MIL-101(Cr)-II	**Figure S4**
(a) TEM image and (b-d) corresponding elemental mapping of MIL-101(Cr)	**Figure S5**
Micropore size distribution of MIL-101(Cr), RHA-MIL-101(Cr)-I, and RHA-MIL-101(Cr)-II calculated from CO_2 adsorption isotherm at 0 ºC	**Figure S6**
Selection of parameter in Autosorb iQ_2 software during the calculation of micropore size distribution of MIL-101(Cr), RHA-MIL-101(Cr)-I, and RHA-MIL-101(Cr)-II calculated from CO_2 adsorption isotherm at 0 ºC	**Figure S7**
Cumulative pore volume of MIL-101(Cr), RHA-MIL-101(Cr)-I, and RHA-MIL-101(Cr)-II calculated from N_2 adsorption isotherm at -196 ºC	**Figure S8**
Dependence of the amount CO_2 adsorbed on the micropore (< 0.7 nm) volume at 0.15 bar and 25 ºC	**Figure S9**
Sips fit of (a) CO_2 and (b) N_2 adsorption isotherms of MIL-101(Cr), RHA-MIL-101(Cr)-I, and RHA-MIL-101(Cr)-II	**Figure S10**
Isosteric heat of adsorption of CO_2 for MIL-101(Cr), RHA-MIL-101(Cr)-I, and RHA-MIL-101(Cr)-II	**Figure S11**
Graphical representation of determination of CO_2 working capacity	**Figure S12**
C and Si content for RHA, MIL-101-Cr, RHA-MIL-101-Cr-I, and RHA-MIL-101-Cr-II	**Table S1**
Fitting parameters and correlation coefficients of Sips model at 25 °C and 1 bar pressure	**Table S2**
Comparative chart for the textural properties and CO_2 adsorption performance of selective MOFs based composites	**Table S3**
Adsorbent's evaluation parameter for VSA process	**Table S4**

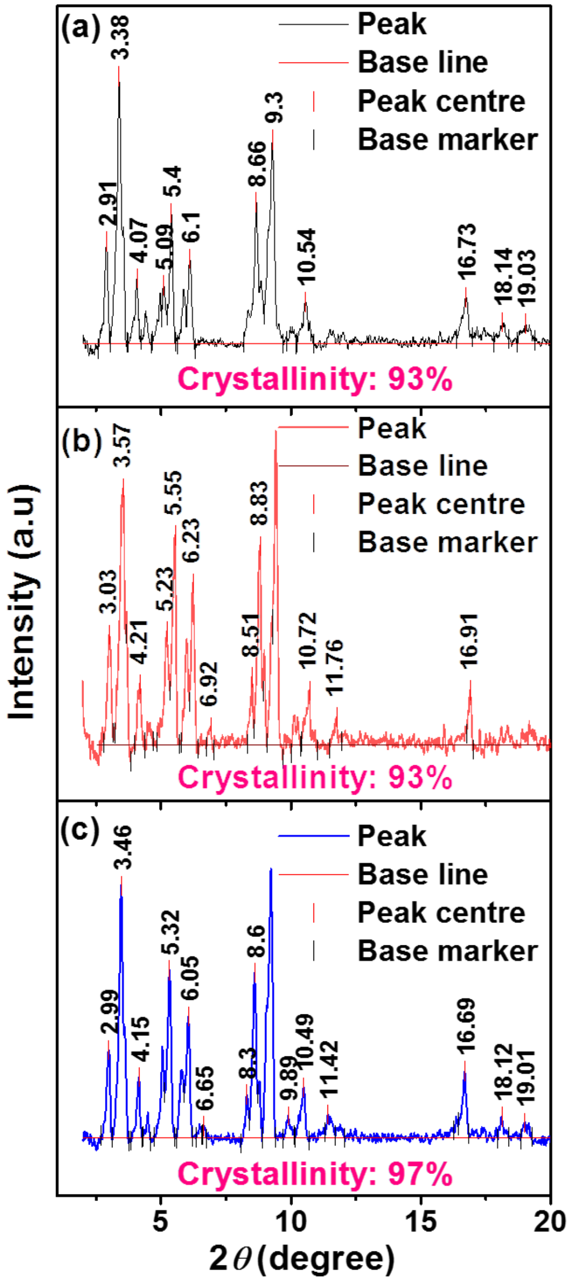

Figure S1. Crystallinity calculation from the P-XRD pattern of (a) MIL-101(Cr), (b) RHA-MIL-101(Cr)-I, and (c) RHA-MIL-101(Cr)-II. (The crystallinity of the synthesized MOFs are calculated from the peak areas from the P-XRD pattern.[S1] Crystallinity (expressed in %) is defined as ratio of remaining diffractogram area due to crystalline peaks by the total area of the original diffractogram.)

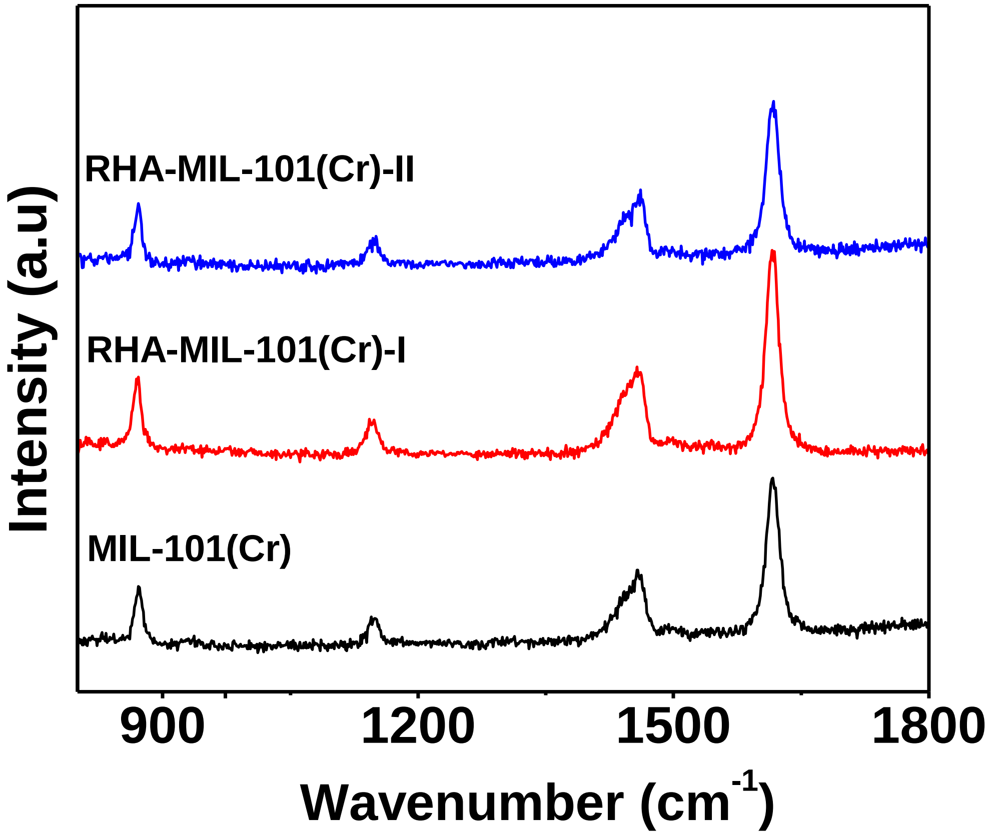

Figure S2. Raman spectra of RHA, MIL-101(Cr), RHA-MIL-101(Cr)-I, and RHA-MIL-101(Cr)-II.

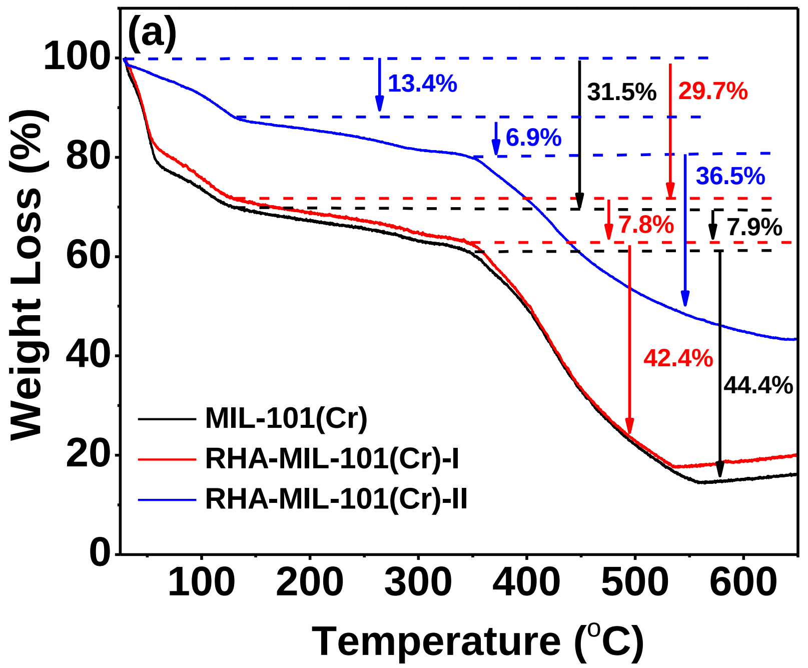

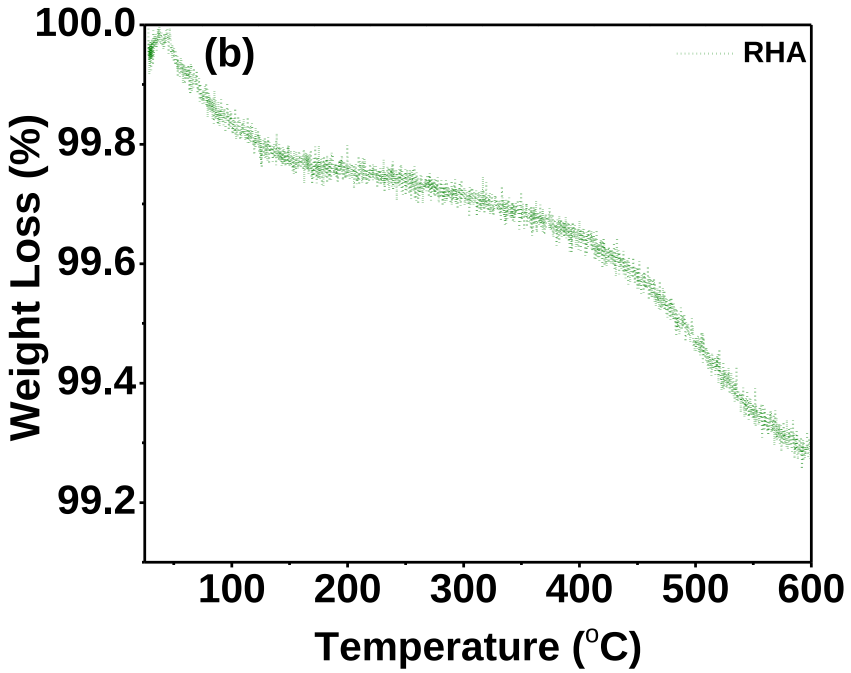

Figure S3. TGA curves of (a) MIL-101(Cr), RHA-MIL-101(Cr)-I, RHA-MIL-101(Cr)-II and (b) RHA.

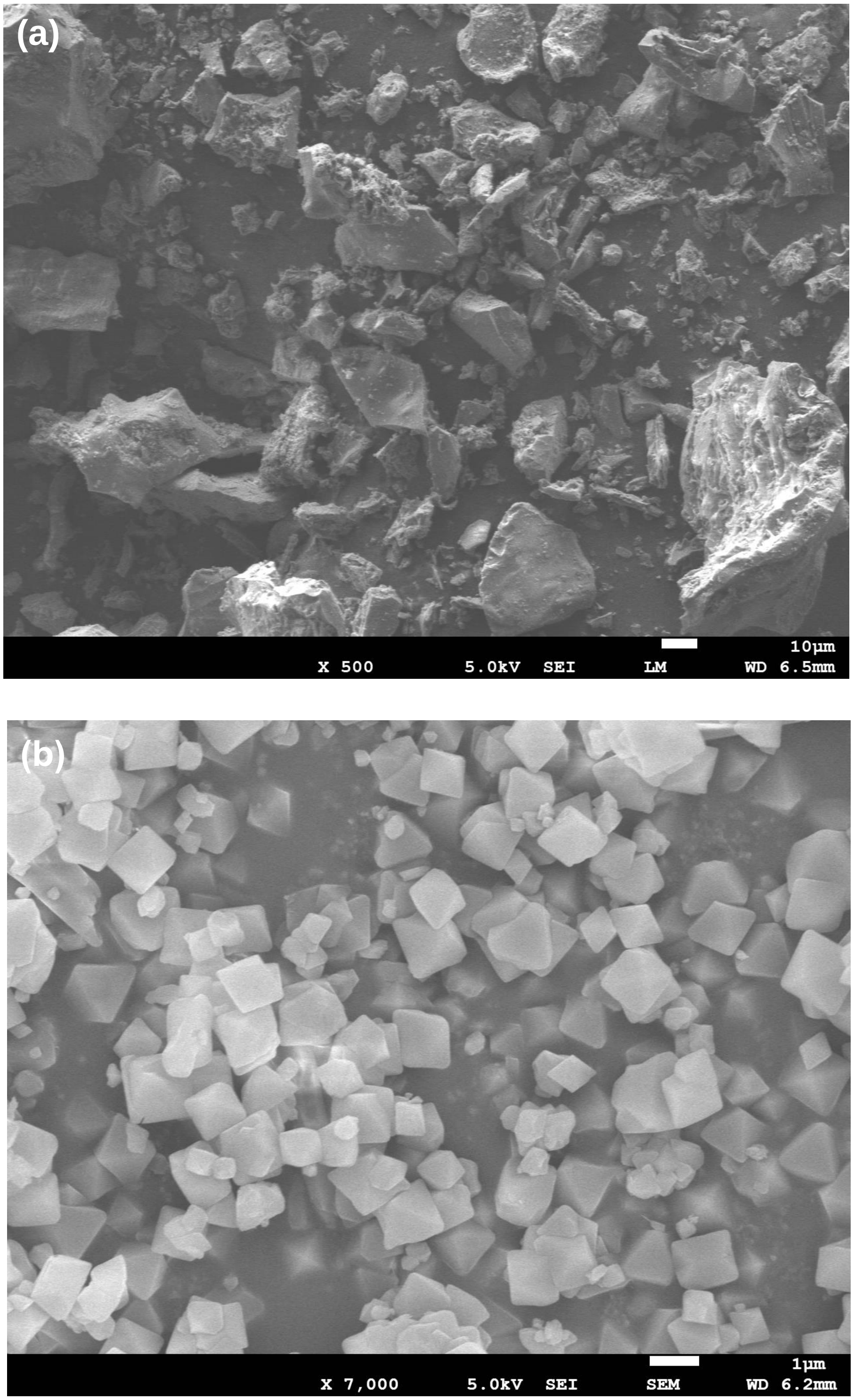
(a)
10μm
X 500 5.0kV SEI LM WD 6.5mm
(b)
1μm
X 7,000 5.0kV SEI SEM WD 6.2mm

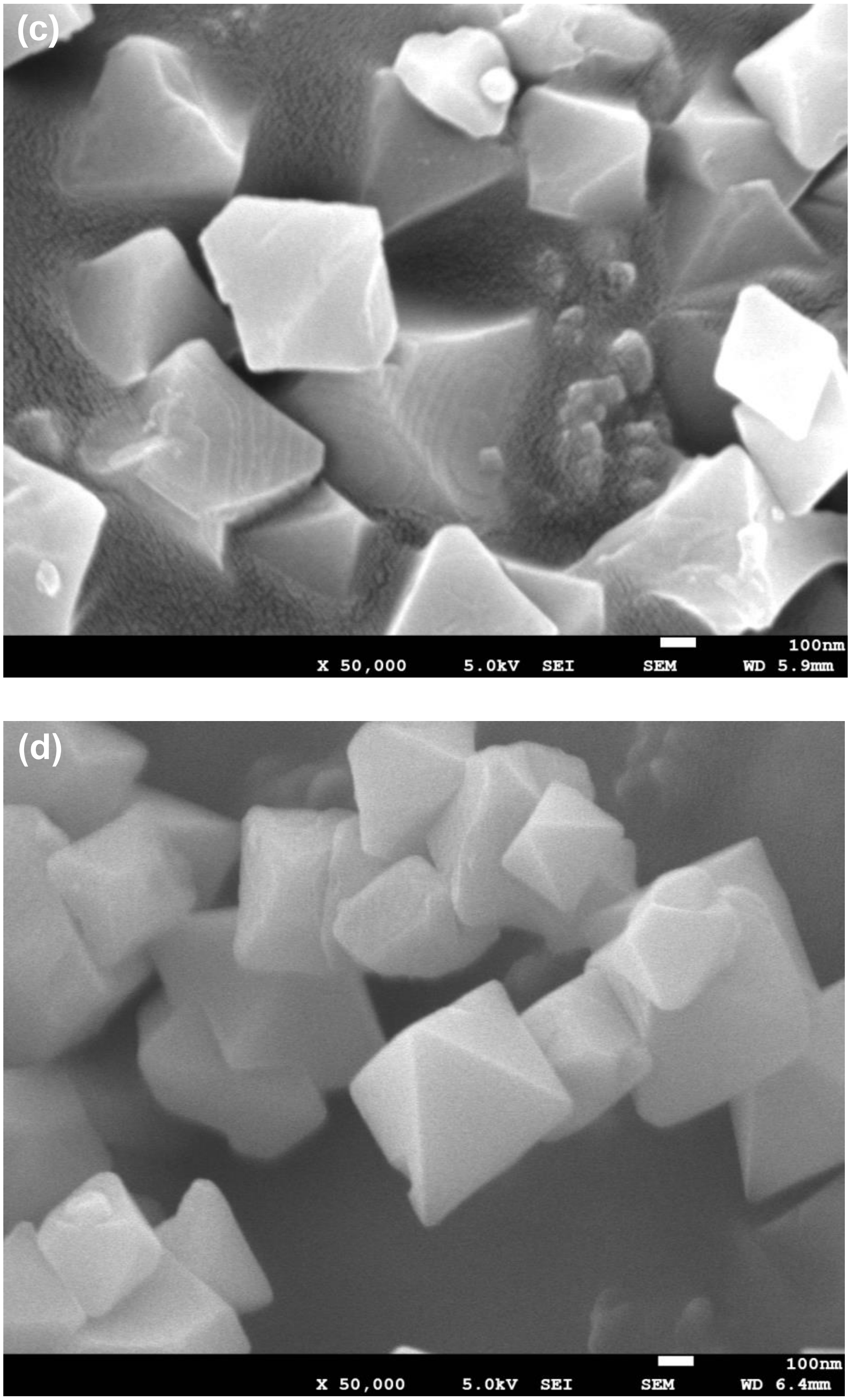

Figure S4. FESEM image of (a) RHA, (b) MIL-101(Cr), (c) RHA-MIL-101(Cr)-I, and (d) RHA-MIL-101(Cr)-II.

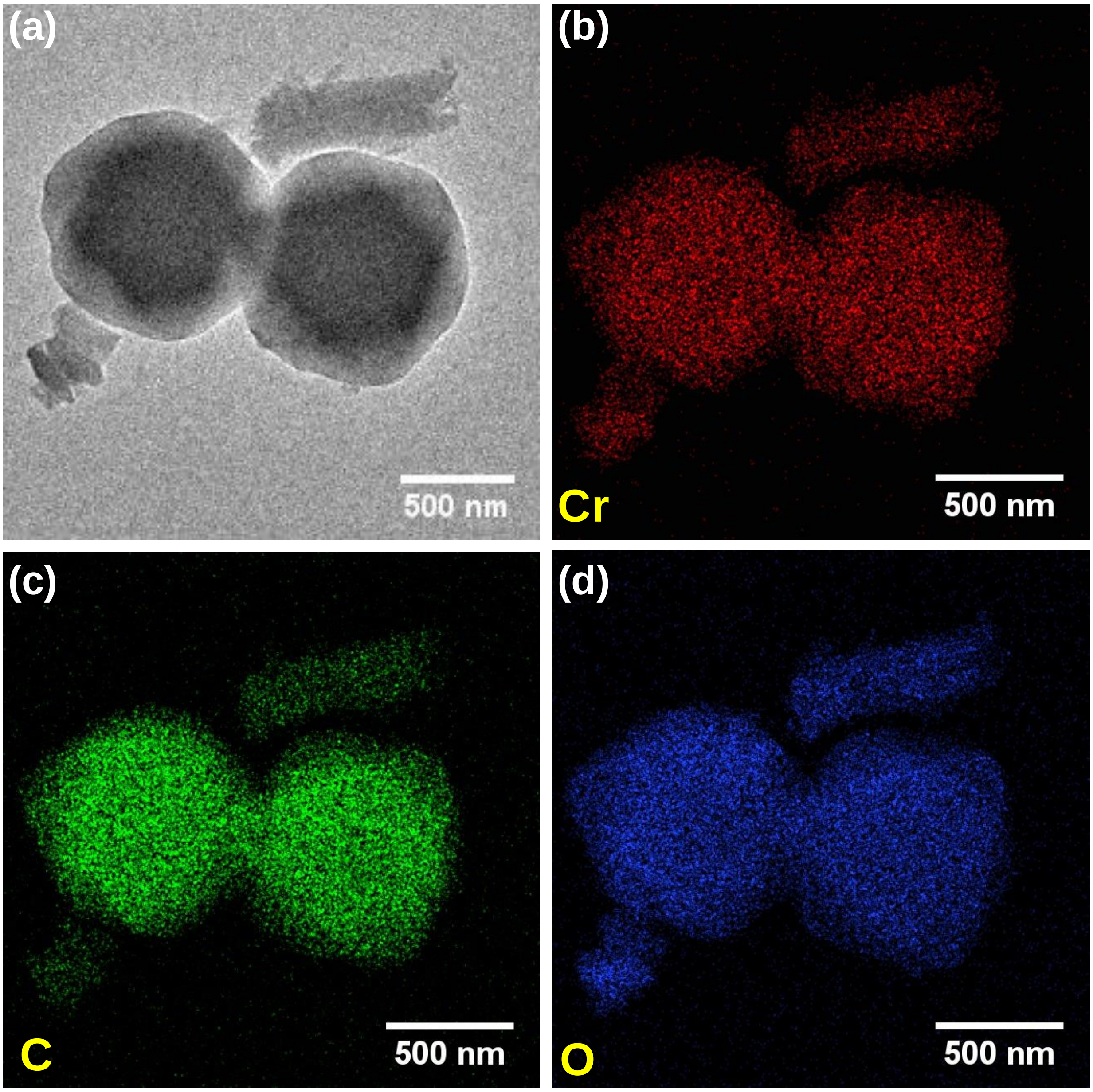

Figure S5. (a) TEM image and (b-d) corresponding elemental mapping of MIL-101(Cr).

Table S1| C and Si content for RHA, MIL-101-Cr, RHA-MIL-101-Cr-I, and RHA-MIL-101-Cr-II.

**Element**	RHA	MIL-101-Cr	RHA-MIL-101-Cr-I	RHA-MIL-101-Cr-II
**C (%)**	8.9	30.6	34.0	37.0
**Si (Atomic %)**	25.3	negligible	0.21	0.23

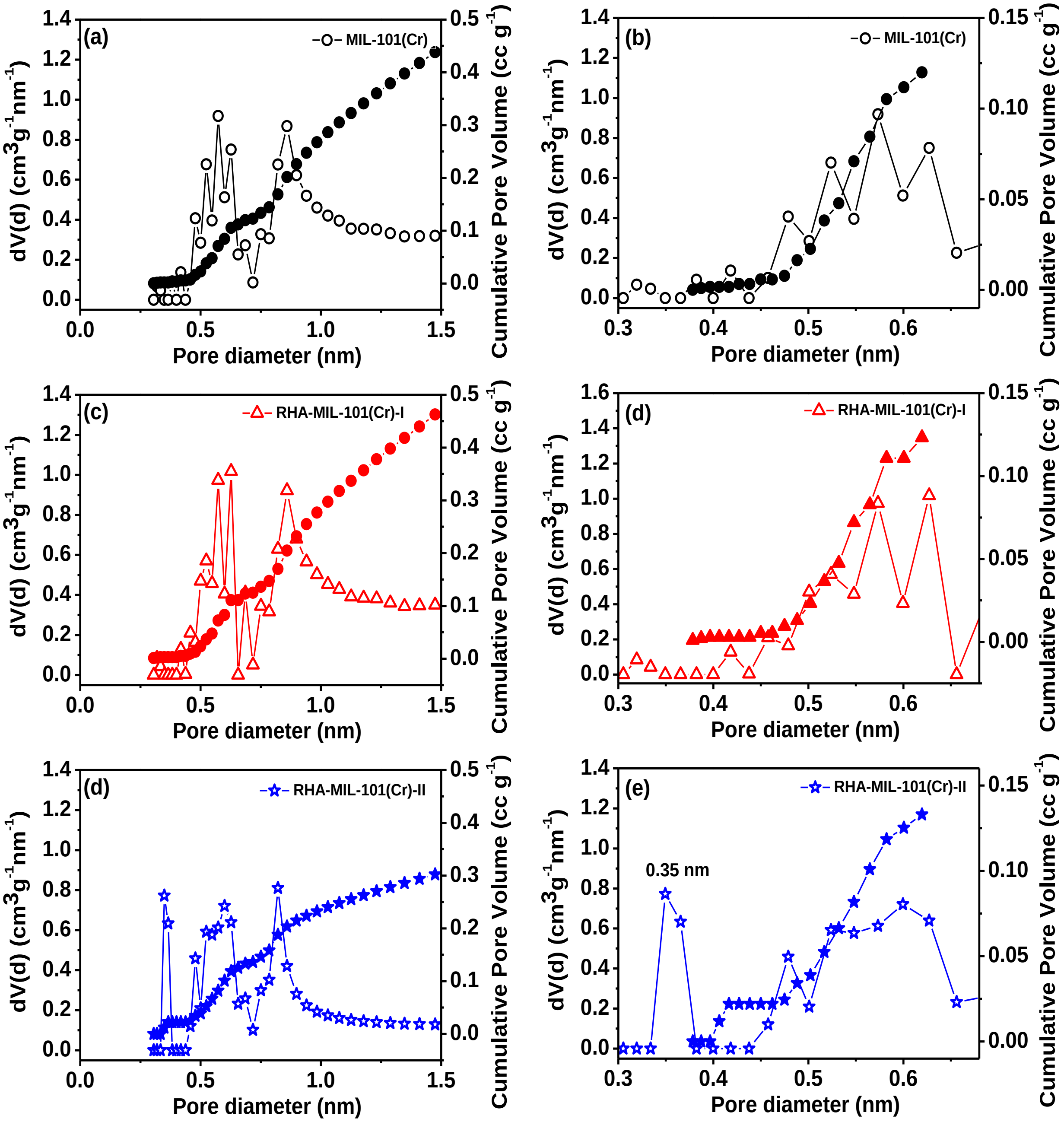

Figure S6. Micropore size distribution of MIL-101(Cr), RHA-MIL-101(Cr)-I, and RHA-MIL-101(Cr)-II calculated from CO_2 adsorption isotherm at 0 ºC.

Calculation of ultra-micropore volume:

Calculation of microporosity by CO_2 at 0 °C is often applied to microporous material like carbon molecular sieve, zeolite, and MOF, due to relatively faster analysis than N_2 at -196 ºC. The method allows determining the pore size distribution of the pores less than 0.4 nm, which is generally difficult using N_2.[S2] The ultra-micropore volume ($V_{ultramicro}$) for the pores < 0.7 nm was calculated using NLDFT method from CO_2 adsorption isotherm at 0 °C with considering carbon adsorbent. The calculation was carried out using Quantachrome ASiQwin^{TM} data processing software equipped along with the instrument.

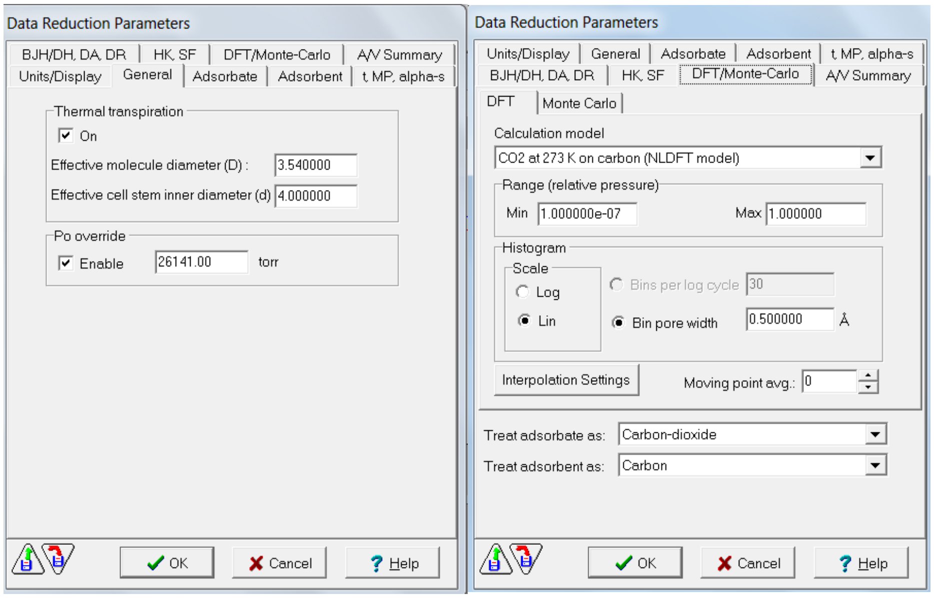

Figure S7. Selection of parameter in Autosorb iQ_2 software during the calculation of micropore size distribution of MIL-101(Cr), RHA-MIL-101(Cr)-I, and RHA-MIL-101(Cr)-II calculated from CO_2 adsorption isotherm at 0 ºC.

During the DFT calculation using software, the first step is to collect the data from CO_2 adsorption isotherm at 0 °C, and then correct the P_0 value to 26141 torr[S3] in data reduction parameter; after the P_0 correction (Figure S7), the P/P_0 scale of the isotherm will automatically be adjusted to a maximum of ~ 0.036. Then set the P/P_0 from 1.000000e^{-07} to 1 in the DFT range of the software with a linear scale (Figure S7) and then generate the fitted graph and exported the

corresponding data. The exported data from the software would come in the pore widths range of 0.3053−1.4748 nm along with corresponding differential pore volume and cumulative pore volume (dV(d)). Among this range, choose the pore width < 0.7 nm (we have taken up to 0.68 nm) and find the ultra-micropore volume from the corresponding cumulative pore volume (cm^3 g^{-1}) and plot the graph using any plotting software such as Origin to get pore size distribution profile.

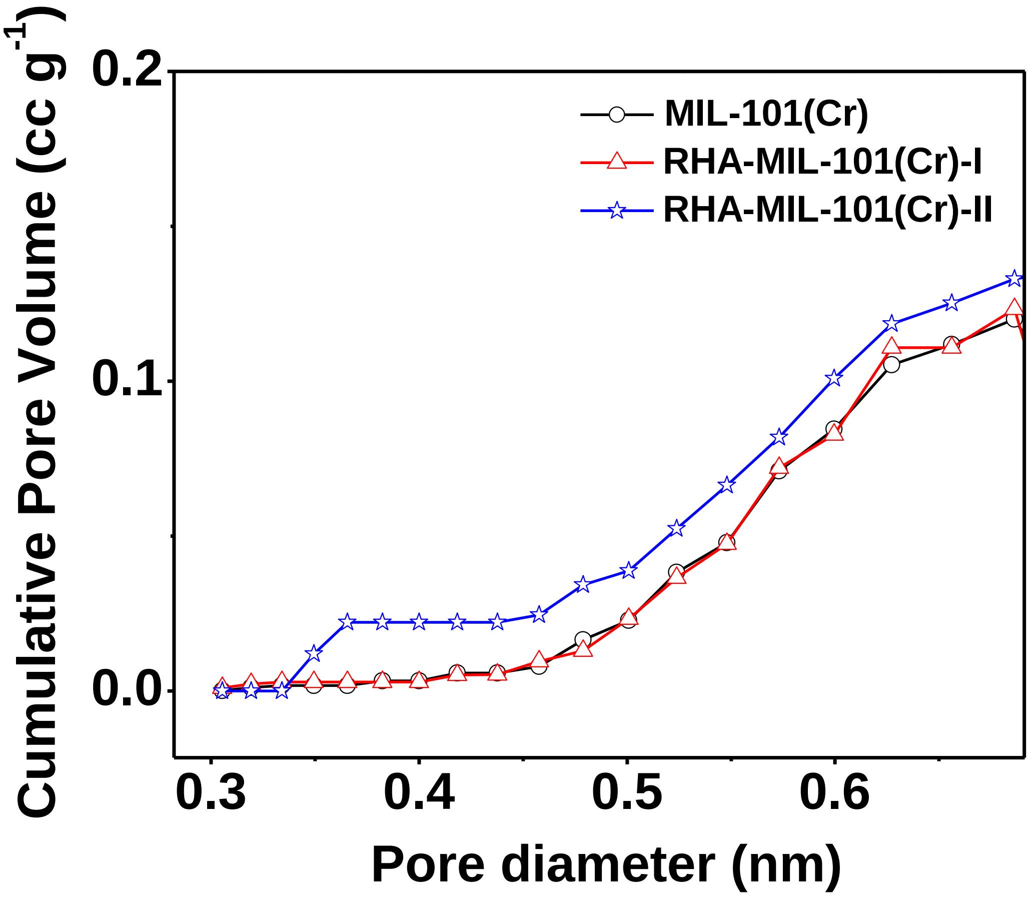

Figure S8. Cumulative pore volume of MIL-101(Cr), RHA-MIL-101(Cr)-I, and RHA-MIL-101(Cr)-II calculated from N_2 adsorption isotherm at -196 ºC.

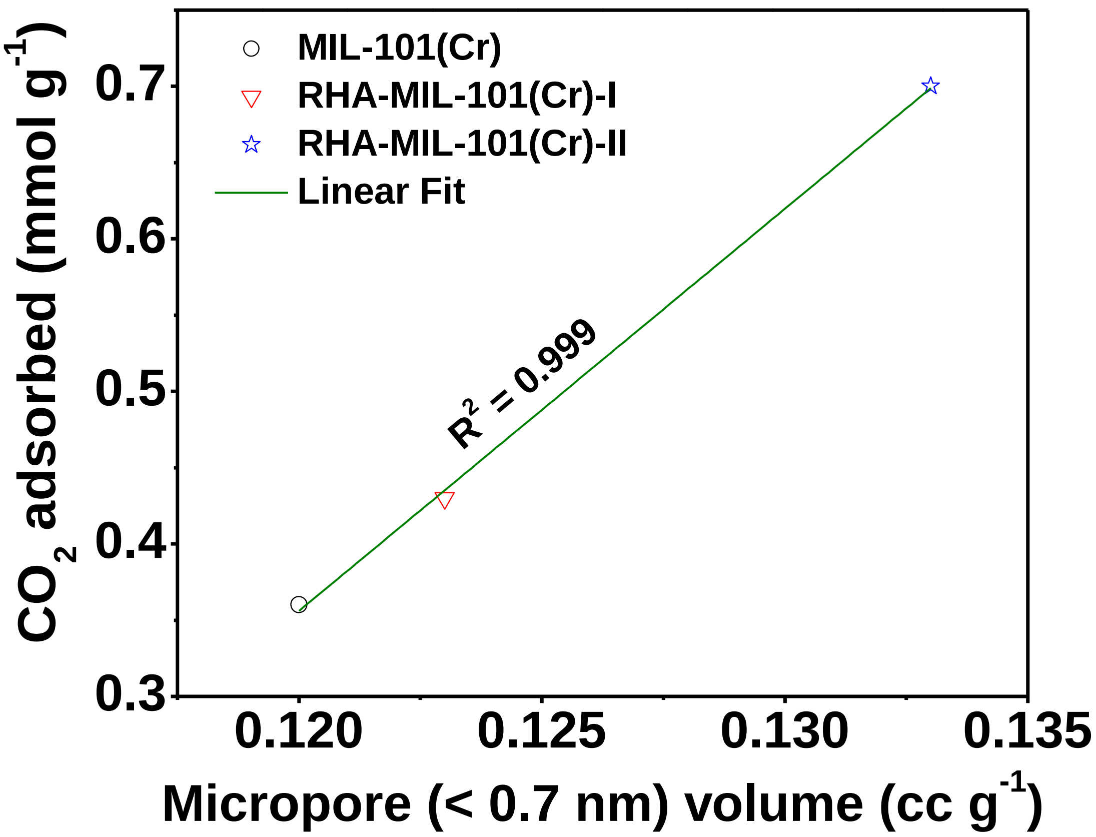

Figure S9. Dependence of the amount CO_2 adsorbed on the micropore (< 0.7 nm) volume at 0.15 bar and 25 ºC.

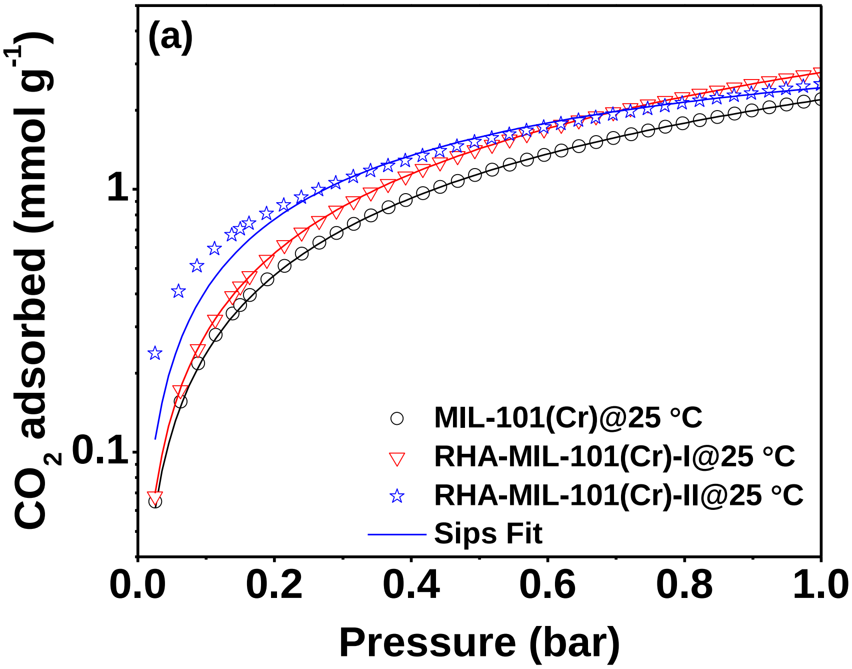

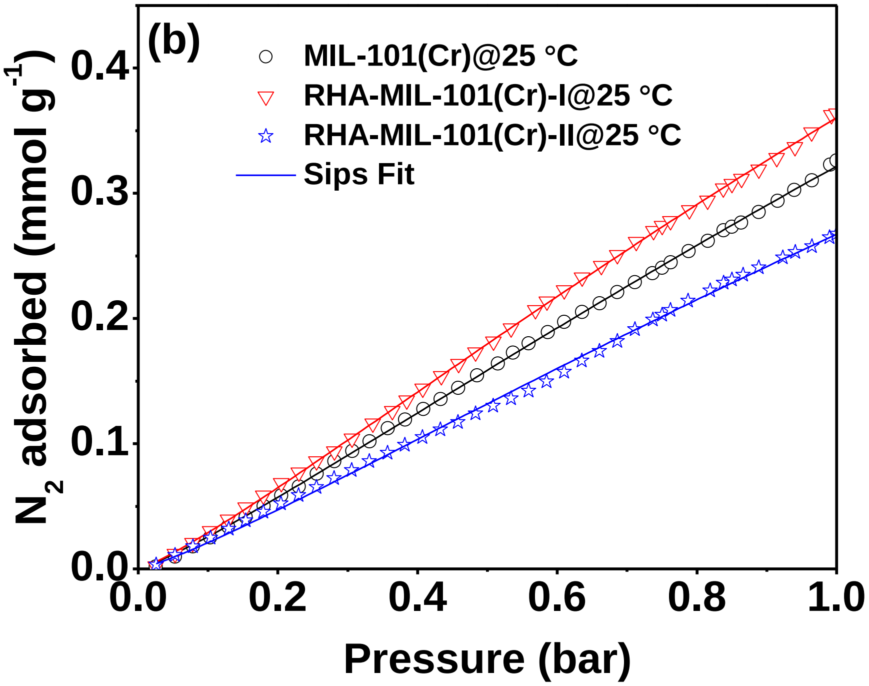

Figure S10. Sips fit of (a) CO_2 and (b) N_2 adsorption isotherms of MIL-101(Cr), RHA-MIL-101(Cr)-I, and RHA-MIL-101(Cr)-II.

Table S2| Fitting parameters and correlation coefficients of Sips model at 25 °C and 1 bar pressure.						
Parameters[a]	MIL-101(Cr)		RHA-MIL-101(Cr)-I		RHA-MIL-101(Cr)-II	
	CO_2	N_2	CO_2	N_2	CO_2	N_2
**q_s (mmol g^{-1})**	25.49	1.69	33.50	1.65	5.33	1.28
**b_s (mmol g^{-1})**	0.09	0.23	0.093	0.27	0.84	0.26
**s**	1	0.84	0.97	0.84	1	0.83
**R^2**	0.999	0.999	0.999	0.999	0.990	0.998

q_s is the saturated adsorption capacity (mmol g^{-1}), and b_s is the affinity constant for Sips model. s characterizes the heterogeneity of the adsorption system and R^2 is the correlation coefficient which indicates the accuracy of the adsorption model.

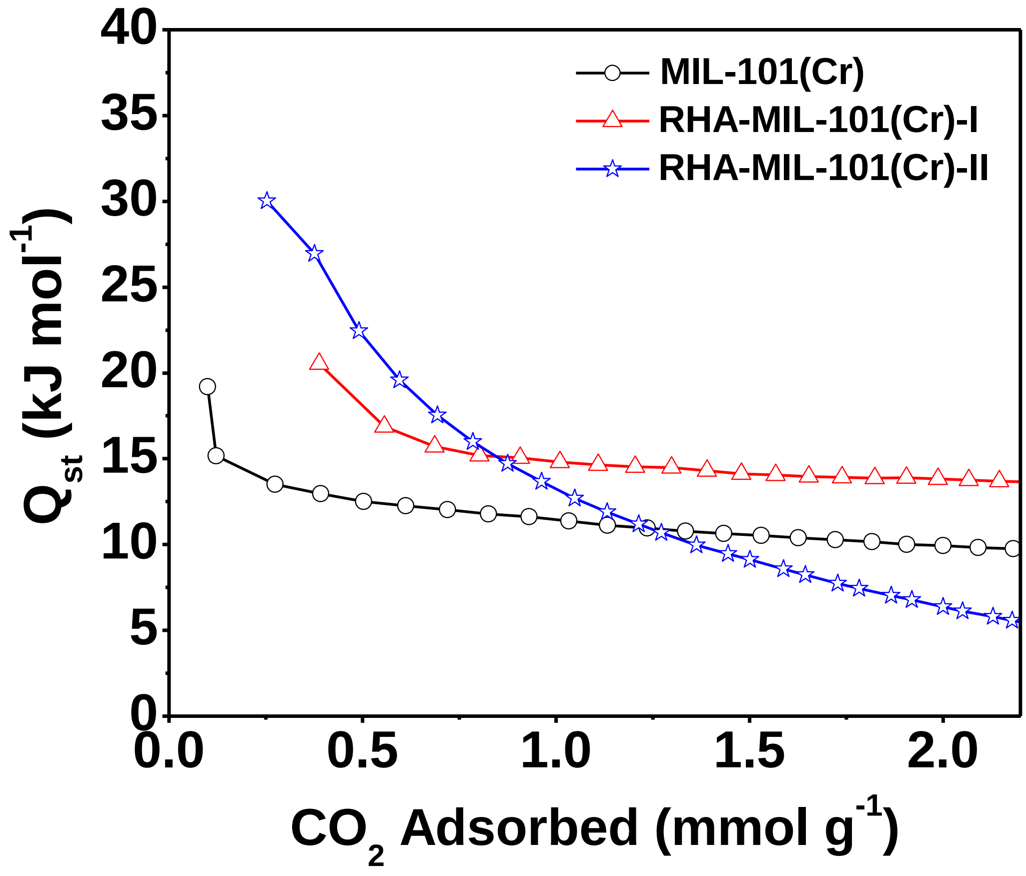

Figure S11. Isosteric heat of adsorption of CO_2 for MIL-101(Cr), RHA-MIL-101(Cr)-I, and RHA-MIL-101(Cr)-II.

Table S3 | Comparative chart for the textural properties and CO_2 adsorption performance of selective MOFs based composites.

Material	Doping (wt %)	S_{BET}[a] ($m^2 g^{-1}$)	V_{total}[b] ($cm^3 g^{-1}$)	Operating conditions		q_{CO2}[d] (mmol g^{-1})	Q_{st}[e] (kJ mol^{-1})	Ref.
				P_{CO_2}[c] (bar)	T (°C)			
Cu (BDC)		489	0.26	1	30	-	-	S4
MCM-41@ Cu (BDC)	3	825	0.64	1<br>4	30<br>30	0.5<br>2.0	-	S4
HKUST-1		1748	0.63	0.1	30	0.96	-	S5
HKUST-1@MCFs	22	1057	1.17	0.1	30	1.40	-	S5
HKUST-1		1611	0.76	1	25	3.35	-	S6
HKUST-1@MCF-NH_2	41.6	1539	0.81	1	25	3.89	-	S6
HKUST		1466	0.60	0.15<br>1	25<br>25	1.05<br>4.16	25	S7
HKUST@SBA-15	1	1745	0.74	0.15<br>1	25<br>25	1.11<br>4.82	46	S7
Mg-MOF-74		-	-	0.15<br>1	20	2.26<br>5.69	-	S8
Mg-MOF-74@SBA-15	57	-	-	0.15<br>1	20	1.56<br>3.93	30	S8
NH_2MIL-53-Al		-	-	1	0	2.7	-	S9
NH_2MIL-53-Al@MSS	40	-	-	1	0	2.0	-	S9
HKUST-1		959	-	2	25	4.25	-	S10
HKUST-1@Natural zeolite	95	-	--	2	25	4.76	-	S10
MOF-74		1180	0.88	1.1	25	5.30	-	S11

MOF-74@Zeolite 5A	5	1504	0.92	1.1	25	7.10	33	S11
ZIF-69		511	-	1	0	0.80	-	S12
ZIF-69@Zeolite 4A	62	470	-	1	0	0.62	25	S12
ZIF-8		1408	0.67	0.15<br>1	0	0.24<br>1.65	32	S13
ZIF-8@GO	10	1414	0.78	0.15<br>1	0	0.35<br>2.19	50	S13
UIO-66		838	0.24	0.15<br>1	25	0.45<br>2.27	-	S14
UIO-66@GO	5	1184	0.38	0.15<br>1	25	1.20<br>3.37	-	S14
Cu(BTC)		1448	0.70	0.15<br>1	25	0.75<br>3.98	-	S15
Cu(BTC)@HCM	27	516	0.26	0.15<br>1	25	1.0<br>2.75	27	S15
MIL-101(Cr)		2235	1.22	1	30	1.28	-	S16
MIL-101(Cr)@Maxorb-III	12.5	2670	1.27	1	30	1.30	-	S16
MIL-101(Cr)		3745	1.95	0.15<br>1	30	0.75<br>3.10	23	S17
MIL-101(Cr)@MWCNT	2.0	3146	1.63	0.15<br>1	30	0.85<br>3.50	27	S17
HKUST		1048	0.51	1	32	0.25	-	S18
HKUST@GO	10.0	1015	0.49	1	32	0.42	-	S18
MIL-101(Cr)		4824	2.41	1	25	1.52	37	S19
MIL-101(Cr)@Meso carbon	4.4	2828	1.55	1	25	2.67	45	S19
Cu(BTC)		892	0.43	1	25	2.32	28	S20

Cu(BTC)@Aminated graphite oxide	10	1367	0.66	1	25	4.65	30	S20
MIL-101(Cr)		3325	1.70	0.15<br>1	25	0.36<br>2.20	19	This work
MIL-101(Cr)@RHA-I	6.25	3744	1.91	0.15<br>1	25	0.43<br>2.79	20	This work
MIL-101(Cr)@RHA-II	12.5	4249	2.27	0.15<br>1	25	0.70<br>2.51	30	This work

[a]BET surface area, [b]Total pore volume at P/P_0 = 0.99, [c]CO_2 adsorption pressure, [d]CO_2 adsorption capacity, [e]Heat of CO_2 adsorption at low surface coverage and - data not found.

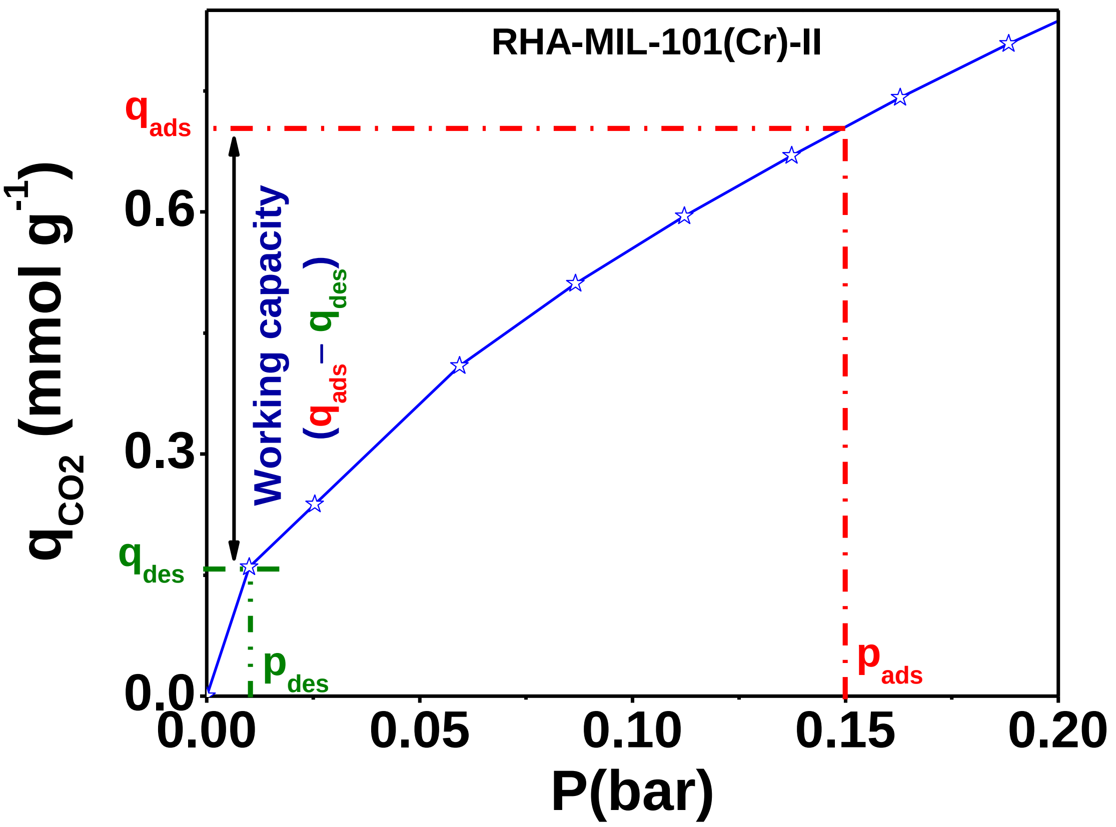

Figure S12. Graphical representation of determination of CO_2 working capacity[S21]

The adsorbent's evaluation parameters were calculated as per the following equations.[S22]

(a) CO_2 working capacity (WC) = q_{CO2}^{ads}- q_{CO2}^{des}

(b) CO_2 over N_2 selectivity ($S_{CO2/N2}$) = (q_{CO2}^{ads}/ q_{N2}^{ads})/ (p_{CO2}^{ads}/ p_{N2}^{ads})

(c) Purity of CO_2 = q_{CO2}^{ads}/ (q_{CO2}^{ads}+ q_{N2}^{ads}) × 100 (%)

where q_{CO2}^{ads}, q_{N2}^{ads} are the amounts of CO_2, and N_2 adsorbed at their respective equilibrium partial pressures (p_{CO2}^{ads} and p_{N2}^{ads}) and q_{CO2}^{des} is the amounts of CO_2 adsorbed at desorption

pressure. In general, for coal-fired power plants, the flue gas was generated at a total pressure of approximately 1 bar having a CO_2 concentration of 15% and an N_2 concentration of 75%.[S23] Under these conditions, the corresponding partial pressure was 0.15 bar (p_{CO2}^{ads}) for CO_2 and 0.75 bar for N_2 (p_{N2}^{ads}). For the VSA process, the CO_2 partial pressure in the adsorption region is 0.15 bar and for desorption is 0.01 bar was considered for this evaluation.[S24]

Table S4| Adsorbent's evaluation parameter for VSA process.

Parameters	Adsorbents		
	MIL-101(Cr)	RHA-MIL-101(Cr)-I	RHA-MIL-101(Cr)-II
**q_{CO2}^{ads} (mmol g^{-1})**	0.36	0.43	0.70
**q_{CO2}^{des} (mmol g^{-1})**	0.02	0.02	0.16
**q_{N2}^{ads} (mmol g^{-1})**	0.24	0.27	0.20
**WC (mmol g^{-1})**	0.34	0.41	0.54
**$S_{CO2/N2}$**	7	8	18
**Purity (%)**	60	61	78

References

S1. Park, S., Baker, J. O., Himmel, M. E., Parilla, P. A., Jonhson, D. K. Cellulose crystallinity index: measurement techniques and their impact on interpreting cellulase performance. *Biotechnol Biofuels* **3**, 1–10 (2010).

S2. Aljamaan, H., Holmes, R., Vishal, V., Haghpanah, R., Wilcox, J., Kovscek, A. R. CO_2 Storage and Flow Capacity Measurements on Idealized Shales from Dynamic Breakthrough Experiments. *Energy Fuels* **31**, 1193−1207 (2017).

S3. Lemmon, E. W., McLinden, M. O. & Friend, D. G. in *Thermophysical Properties of Fluid Systems* (eds. Linstrom, P.J., Mallard, W.G.) NIST Chemistry WebBook, NIST Standard Reference Database Number 69, (National Institute of Standards and Technology, Gaithersburg MD, 20899). https://doi.org/10.18434/T4D303 (2018).

S4. Tari, N. E., Tadjarodi, A., Tamnanloo, J. & Fatemi, S. One pot microwave synthesis of MCM-41/Cu based MOF composite with improved CO_2 adsorption and selectivity. *Microporous Mesoporous Mater.* **231**, 154–162 (2016).

S5. Xin, C. *et al.* Enhanced CO_2 Adsorption Capacity and Hydrothermal Stability of HKUST-1 via Introduction of Siliceous Mesocellular Foams (MCFs). *Ind. Eng. Chem. Res.* **55**, 7950–7957 (2016).

S6. Zhu, C. *et al.* Synthesis of HKUST-1#MCF compositing materials for CO_2 adsorption. *Microporous Mesoporous Mater.* **226**, 476–481 (2016).

S7. Chen, C. *et al.* Synthesis of Hierarchically Structured Hybrid Materials by Controlled Self-Assembly of Metal-Organic Framework with Mesoporous Silica for CO_2 Adsorption. *ACS Appl. Mater. Interfaces* **9**, 23060–23071 (2017).

S8. Chakraborty, A. & Maji, T. K. Mg-MOF-74@SBA-15 hybrids: Synthesis, characterization,and adsorption properties. *APL Mater.* **2**, 124107 (2014).

S9. Sorribas, S. *et al.* Synthesis and gas adsorption properties of mesoporous silica-NH_2-MIL-53(Al) core-shell spheres. *Microporous Mesoporous Mater.* **225**, 116–121 (2016).

S10. Lestari, W. W. *et al.* Composite Material Consisting of HKUST-1 and Indonesian Activated

Natural Zeolite and its Application in CO_2 Capture. Open Chem. **17**, 1279–1287 (2019).

S11. Al-Naddaf, Q., Thakkar, H. & Rezaei, F. Novel Zeolite-5A@MOF-74 Composite Adsorbents with Core-Shell Structure for H_2 Purification. *ACS Appl. Mater. Interfaces* **10**, 29656–29666 (2018).

S12. Aldrich, J. H., Rousselo, S. M., Yang, M. L., Araiza, S. M. & Tian, F. Adsorptive Separation of Methane from Carbon Dioxide by Zeolite@ZIF Composite. *Energy Fuels* **33**, 348–355 (2019).

S13. Chen, B., Zhu, Y. & Xia, Y. Controlled in situ synthesis of graphene oxide/zeolitic imidazolate framework composites with enhanced CO_2 uptake capacity. *RSC Adv.* **5**, 30464–30471 (2015).

S14. Cao, Y., Zhao, Y., Lv, Z., Song, F. & Zhong, Q. Preparation and enhanced CO_2 adsorption capacity of UiO-66/graphene oxide composites. *J. Ind. Eng. Chem.* **27**, 102–107 (2015).

S15. Qian, D., Lei, C., Hao, G. P., Li, W. C. & Lu, A. H. Synthesis of hierarchical porous carbon monoliths with incorporated metal-organic frameworks for enhancing volumetric based CO_2 capture capability. *ACS Appl. Mater. Interfaces* **4**, 6125–6132 (2012).

S16. Kayal, S. & Chakraborty, A. Activated carbon (type Maxsorb-III) and MIL-101(Cr) metal organic framework based composite adsorbent for higher CH_4 storage and CO_2 capture. *Chem. Eng. J.* **334**, 780–788 (2018).

S17. Qasem, N. A. A., Qadir, N. U., Ben-Mansour, R. & Said, S. A. M. Synthesis, characterization, and CO_2 breakthrough adsorption of a novel MWCNT/MIL-101(Cr) composite. *J. CO_2 Util.* **22**, 238–249 (2017).

S18. Zhao, Y., Cao, Y. & Zhong, Q. CO_2 Capture on Metal-Organic Framework and Graphene Oxide Composite Using a High-Pressure Static Adsorption Apparatus. *J. Clean Energy Technol.* **2**, 34–37 (2014).

S19. Zhang, Z. *et al.* Chromium-based metal–organic framework/mesoporous carbon composite: synthesis, characterization and CO_2 adsorption. *Adsorption* **21**, 77–86 (2015).

S20. Policicchio, A., Zhao, Y., Zhong, Q., Agostino, R. G. & Bandosz, T. J. Cu-BTC/aminated graphite oxide composites as high-efficiency CO_2 capture media. *ACS Appl. Mater. Interfaces*

6, 101–108 (2014).

S21. Tao, L., Xiao, P., Qader, A., Webley P. A. CO_2 capture from high concentration CO_2 natural gas by pressure swing adsorption at the CO2CRC Otway site, Australia. *Int. J. Greenh. Gas Control.* **83**, 1–10 (2019).

S22. Bae, Y. S. & Snurr, R. Q. Development and evaluation of porous materials for carbon dioxide separation and capture. *Angew. Chem. Int. Ed.* **50**, 11586–11596 (2011).

S23. D'Alessandro, D.M., Smit, B. & Long, J.R. Carbon dioxide capture: prospects for new materials. *Angew. Chem. Int. Ed*. **49**, 6058–6082 (2010).

S24. Liu, Q., Pham, T., Porosoff, M. D. & Lobo, R. F. ZK-5: A CO_2 -Selective Zeolite with High Working Capacity at Ambient Temperature and Pressure. *ChemSusChem* **5**, 2237–2242 (2012).
